# Spin dependent thermoelectric transport in a multiterminal quantum dot hybrid including a superconductor and ferromagnets

**DOI:** 10.1038/s41598-025-94991-2

**Published:** 2025-04-25

**Authors:** Vrishali Sonar, Piotr Trocha

**Affiliations:** Institute of Spintronics and Quantum Information, Faculty of Physics and Astronomy, Adam Mickiewicz University, Poznan, 61-614 Poland

**Keywords:** Physics, Condensed-matter physics, Spintronics

## Abstract

We investigate the thermoelectric response of a hybrid system consisting of two ferromagnetic electrodes and one superconducting lead coupled to a single-level quantum dot with finite Coulomb repulsion. Using the non-equilibrium Green’s function technique within the Hubbard-I approximation, local and non-local thermoelectric coefficients, along with their spin counterparts, such as electrical and thermal conductance, and the Seebeck coefficient are calculated up to linear order with respect to generalized forces. Here, we present a derivation of spin-dependent thermoelectric coefficients for a three-terminal system, extending the existing theory which allowed to describe only cases independent of spin-bias voltage, i.e. when spin accumulation is irrelevant. In the considered system, four competing processes- single particle tunneling, quasiparticle tunneling, direct and crossed Andreev reflection make the system highly adaptable for tuning charge and heat currents. A full analysis of their impact on thermoelectric effects is provided. Moreover, the output power and efficiency of the system operating as a heat engine are evaluated. The extensive goal of this work is to demonstrate how the presence of an additional terminal modifies the hybrid QD-based device’s performance and under which conditions non-local thermoelectric effects become significant.

## Introduction

The contemporary interest and necessity in energy harvesting have made research thrive in various directions of the same and led to ideas of designing systems and materials with elevated thermoelectric efficiency. Achieving this has been proved to be easier in the mesoscopic systems^1^. In the low-dimensional transport systems, the multi-terminal *hybrids*, i. e. an ensemble of a quantum dot coupled to electrodes of characteristic materials are recently becoming desirable by the virtue of their potential applications and providing an arena to understand fundamental physics. When at least one electrode is superconducting (SC), the direct Andreev and cross Andreev transport become possible^2,3^ along with normal electron tunneling. This forms the basis of one of hybrids’ applications, i. e. as Cooper pair splitter and, consequently as spin filtering devices and studying the entanglement properties of non-local electrons^4–11^. The achieved experimental Cooper pair splitting efficiency is around $60\%$^12^, while theoretical conjecture is greater than $90\%$^9,10^ and advocates for parameter tuning to increase cross Andreev reflection and better control of tunneling in and out of the quantum dot.

Such an ensemble can potentially provide a way to overcome the poor thermal conductivity of the superconductor while exploiting its high electrical conductance^13^. Recent works have studied the charge and heat current contributions due to various kinds of tunneling processes when one of the leads is superconductor^14–16^.Better tunability and efficiency are prima facie expected from multi-terminal hybrids. In this scenario, the non-local effects are obviously expected. However, the definition of thermoelectric coefficients is not unique when more than two leads are present, and many definitions of non-local coefficients have been proposed depending on the constraints of generalized forces^17–22^. The generic formulation given by Mazza *et al*.^22^ is exhaustive in defining the electrical and thermal conductance and naturally leads to two terminal relations of thermoelectric coefficients. This recently has been adopted for superconductor-normal metal system^23,24^ and theoretically revealed the possibility to control heat and charge current separately^25^.

Multi-terminal hybrids endow applicability both as a heat engine‘^25–30^ or refrigerator^31–33^ depending on the systems’ parameters configuration. Some experimental realizations have already been demonstrated^34–36^. However, additional theoretical studies are necessary to optimize the relationship between output power and the associated efficiency.

The situation becomes more interesting when some of the leads in a multi-terminal hybrid system are ferromagnetic (FM), which encourages further investigation in the field of spin caloritronics^37^. Finite spin polarization is useful for tuning tunneling phenomena, such as Andreev processes and single-electron tunneling. Estimating the entanglement between split electrons^38,39^ is crucial, highlighting the desirability of a comprehensive study on the transport properties of DQD Cooper pair splitters with ferromagnetic contacts^40,41^. The variation in spin density within ferromagnetic electrodes enables dual control over the system’s magnetic properties-both by adjusting the polarization of the leads and tuning the relative magnetization angle between the two electrodes. Additionally, other intrinsic system parameters can induce qualitative changes in transport properties leading to behaviors distinct from those observed in two-terminal devices. A comprehensive characterization of these systems is still lacking, motivating us to address this problem.

Thus, the aim of the present studies is twofold: (i) To describe local and non-local conventional thermoelectric coefficients and their spin counterparts in a hybrid consisting of two ferromagnetic and one superconducting lead coupled to a quantum dot (QD) viz. electrical and thermal conductance, Seebeck coefficients and output power in both the linear and non-linear response regimes. (ii) To evaluate the prospects of the system working as a heat engine and examine the trade-off between power and efficiency. The obtained results clearly indicate the advantage of the 3-terminal system compared to 2-terminal QD-based hybrids operating as heat engines. Moreover, the thermoelectric properties can be effectively tuned by modifying the magnetic configuration of the ferromagnetic electrodes-a feature unattainable in two-terminal hybrids but crucial for potential applications in computational technologies.

We point out that current experimental techniques^42^ enable the realization of the considered system, where switching between parallel and antiparallel magnetic configurations is achieved by applying an external magnetic field^43–45^. Therefore, the present work can pave the way for designing relevant experiments and assist in interpreting the results obtained.

The content of the article is designed as follows: Sec. Theoretical description includes theoretical description of the model followed by the charge and heat currents definition and formulation, algebraic forms of local and non-local thermoelectric coefficients, spin dependent coefficients as well as power and efficiency formulae. Sec. Numerical results and discussions shows numerical results in the linear response regime, and further in Sec. Non-linear response regime, the performance parameters for hybrid working as a heat engine are presented while charge and heat currents are calculated in non-linear regime. Finally, Sec. Summary concludes results.

**Fig. 1:**
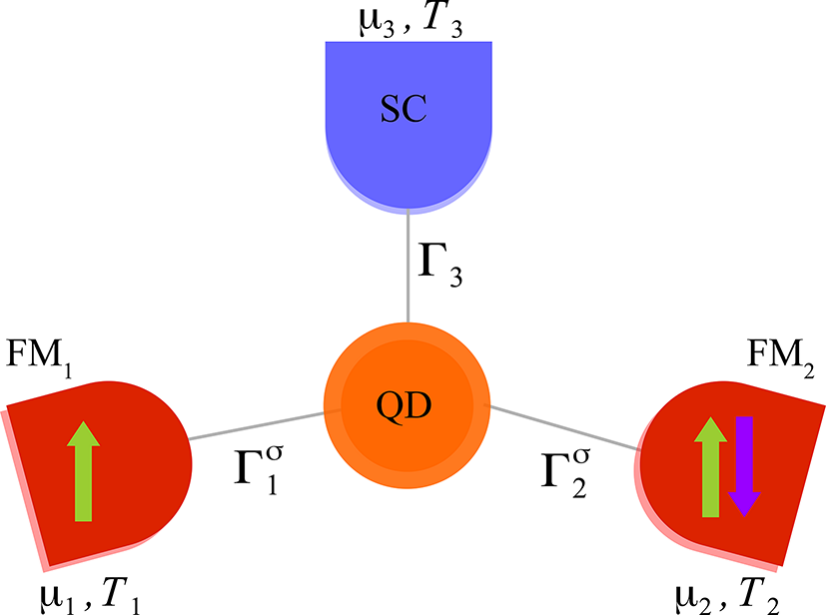
Scheme of the considered model. A single level quantum dot (QD) is tunnel coupled with two ferromagnetic leads, $\text{FM}_1$ and $\text{FM}_2$, and with superconductor (SC). $\Gamma _i^\sigma$ ($\Gamma _3$) denotes coupling strength to $i=1,2$th ferromagnetic (superconducting) electrode. Each electrode can be characterised by its own chemical potential ($\mu _i$) and temperature ($T_i$). Thick arrows denote direction of magnetizations of the ferromagnets: green (green and purple) arrows indicate parallel (antiparallel) magnetic alignment.

## Theoretical description

### Model

The model device taken under consideration, as depicted in Fig. 1, consists of three bulk leads: two ferromagnetic and one superconducting. All of them are tunnel coupled to a single-level quantum dot. The general Hamiltonian describing the system can be formulated using the single-impurity Anderson model and the BCS model for *s-wave* superconducting lead in the second quantization formalism, as follows,

1 $$\begin{aligned} H=H_{FM}+H_{SC}+H_{QD}+H_{T}. \end{aligned}$$

The first term, $H_{FM}$, describes the ferromagnetic leads and is modeled by

2 $$\begin{aligned} H_{FM}=\sum _{{\textbf{k}}i\sigma } \varepsilon _{{\textbf{k}}i\sigma } c_{{\textbf{k}}i\sigma }^{\dagger }c_{{\textbf{k}}i\sigma } , \end{aligned}$$

where $i=1, 2$ for either ferromagnetic lead. Here, $\varepsilon _{{\textbf{k}}i\sigma}$ denotes the spin-dependent single electron energy for spin $\sigma$ and wave vector ${\textbf{k}}$ in the *i*th ferromagnetic lead. Next is the Hamiltonian for *s-wave* superconducting lead within the BCS approximation and is given by,

3 $$\begin{aligned} H_{SC}=\sum _{{\textbf{k}}\sigma } \varepsilon _{{\textbf{k}}3\sigma } c_{{\textbf{k}}3\sigma }^{\dagger }c_{{\textbf{k}}3\sigma } + \Delta \sum _{{\textbf{k}}} ( c_{{\textbf{k}}3\downarrow }c_{-{\textbf{k}}3\uparrow } +c_{-{\textbf{k}}3\uparrow }^{\dagger } c_{{\textbf{k}}3\downarrow }^{\dagger }), \end{aligned}$$

with $\varepsilon _{{\textbf{k}}3\sigma }$ denoting relevant single electron energy in the superconductor. $\Delta$ is the superconducting energy gap, assumed to be real. Furthermore, $H_{QD}$ is the Hamiltonian for a quantum dot with $\varepsilon _d$ being a dot’s level energy, whereas the relevant Coulomb repulsion energy of the pair of electrons of opposite spin residing in QD’s level is parameterized by *U* and acquires the form

4 $$\begin{aligned} H_{QD}=\sum _{\sigma } \varepsilon _{d} d_{\sigma }^{\dagger }d_{\sigma } +Un_{\downarrow }n_{\uparrow } \end{aligned}$$

The particle tunneling between QD and *i*th lead is described by the corresponding tunneling Hamiltonian,

5 $$\begin{aligned} H_{T}=\sum _{{\textbf{k}}i\sigma } V_{{\textbf{k}}i\sigma } c_{{\textbf{k}}i\sigma }^{\dagger }d_{\sigma } + \mathrm{H.c.} \end{aligned}$$

Here, H.c. denotes the Hermitian conjugate of the first term in $H_{T}$ and $V_{{\textbf{k}}i\sigma }$ being *spin conserving* tunneling matrix elements. In the wide band limit, these elements are parameterized in terms of energy-independent coupling matrices for ferromagnetic leads ($i=1,2$) as $\Gamma _{i\sigma }= 2\pi \left\langle |V_{{\textbf{k}}i\sigma }|^{2}\right\rangle \rho _{i\sigma }$ where $\rho _{i\sigma }$ is the spin-dependent density of states in the *i*th FM lead and $\langle \dots \rangle$ denotes the average over ${\textbf{k}}$^41^ . Introducing spin polarization factor $p_i$ for the *i*th FM lead, the corresponding coupling can be expressed as $\Gamma _{i}^{\sigma }=\Gamma _{i}(1\pm p_i)$ with upper sign for $\sigma =\uparrow$ and lower sign for $\sigma =\downarrow$. Here, $\Gamma _{i}=(\Gamma _{i}^{\uparrow }+\Gamma _{i}^{\downarrow })/2$.

The equation of motion for the retarded/advanced Green’s function is solved with respect to the Hamiltonian Eq. (1) within Hubbard-I approximation. In Nambu space^46^, the matrix of the retarded Green function, written in the Zubarev notation^47^ can be given as ${\textbf{G}}^r= \langle \langle {\textbf{X}}^{\dagger }| {\textbf{X}}\rangle \rangle$ with ${\textbf{X}}= (d_\uparrow ^\dagger , d_\downarrow , d_\downarrow ^\dagger , d_\uparrow )$. The advanced Green’s function is obtained using relation ${\textbf{G}}^a=[{\textbf{G}}^r]^\dag$. Detailed form of the Green’s function is presented in the Supplementary Information. The coupling matrix for FM leads takes diagonal form for parallel (antiparallel) alignment of their magnetic moments, $\varvec{\Gamma }_1=\text{diag}(\Gamma _{1}^\uparrow , \Gamma _{1}^\downarrow , \Gamma _{1}^\downarrow , \Gamma _{1}^\uparrow )$ and $\mathbf {\Gamma }_2=\text{diag}(\Gamma _{2}^{\uparrow (\downarrow )}, \Gamma _{2}^{\downarrow (\uparrow )}, \Gamma _{2}^{\downarrow (\uparrow )},\Gamma _{2}^{\uparrow (\downarrow )})$. In turn, the dot’s coupling to the SC lead has the following form:

6 $$\begin{aligned} \mathbf {{\Gamma }_{3}}=\Gamma _3{\rho }_R(\varepsilon )\begin{pmatrix} 1 & -\frac{\Delta }{\varepsilon } & 0 & 0\\ -\frac{\Delta }{\varepsilon } & 1 & 0 & 0\\ 0 & 0 & 1 & \frac{\Delta }{\varepsilon } \\ 0 & 0 & \frac{\Delta }{\varepsilon } & 1 \end{pmatrix} \end{aligned}$$

with

7 $$\begin{aligned} {\rho }_R(\varepsilon ) =\frac{|\varepsilon |\theta (|\varepsilon |-\Delta )}{\sqrt{\varepsilon ^{2}-\Delta ^{2}}} \end{aligned}$$

denoting the density of states of the superconductor and $\Gamma _3$ represents the coupling of the dot to the superconducting electrode in the normal state.

The retarded self-energy matrix due to the coupling between *i*th ferromagnetic lead and quantum dot is given as^48^, $\mathbf {\Sigma }_{i}^r=-\frac{i}{2}\mathbf {\Gamma }_i$ ($i=1,2$), while that due to coupling between the superconductor and QD is expressed by

8 $$\begin{aligned} \mathbf {\Sigma }_{3}^r = -\frac{i}{2}\Gamma _{3}{\tilde{\rho }}_R(\varepsilon ) \begin{pmatrix} 1 & -\frac{\Delta }{\varepsilon } & 0 & 0\\ -\frac{\Delta }{\varepsilon } & 1 & 0 & 0\\ 0 & 0 & 1 & \frac{\Delta }{\varepsilon } \\ 0 & 0 & \frac{\Delta }{\varepsilon } & 1\\ \end{pmatrix}, \end{aligned}$$

where ${\bar{\rho }}_R(\varepsilon )$ is a modified BCS density of states in the superconductor and acquires the form

9 $$\begin{aligned} {\tilde{\rho }}_R(\varepsilon ) =\frac{|\varepsilon |\theta (|\varepsilon |-\Delta )}{\sqrt{\varepsilon ^{2}-\Delta ^{2}}}-i\frac{\varepsilon \theta (\Delta -|\varepsilon |)}{\sqrt{\Delta ^{2}-\varepsilon ^{2}}}. \end{aligned}$$

The lesser self-energy for *i*th electrode ($i=1,2,3$) can be obtained using the relation

10 $$\begin{aligned} \mathbf {\Sigma }_{i}^< = {\textbf{F}}_i(\mathbf {\Sigma }_{i}^a-\mathbf {\Sigma }_{i}^r). \end{aligned}$$

Here, ${\textbf{F}}_i=\text{diag}(f_{i}^{-}, f_{i}^{+}, f_{i}^{-}, f_{i}^{+})$ and $f_i^{\pm }= [1+\exp (\varepsilon \pm \mu _i)/k_bT_{i}]^{-1}$ is the Fermi-Dirac distribution function with $\mu _i$ being the chemical potential for a given lead. The total lesser self-energy is given as $\mathbf {\Sigma }^{r/a} = \mathbf {\Sigma }_1^{r/a}+\mathbf {\Sigma }_2^{r/a}+\mathbf {\Sigma }_{3}^{r/a}$. Finally, the lesser Green’s function is obtained by using the Keldysh relation,

11 $$\begin{aligned} {\textbf{G}}^<={\textbf{G}}^r\mathbf {\Sigma }^<{\textbf{G}}^a \end{aligned}$$

with $$\mathbf {\Sigma }^{<} = \sum _i\mathbf {\Sigma }_i^{<}$$ for $$i=1,2,3$$.

### Charge and heat current

The current flowing out of the *i*th ($i=1,2$) lead can be expressed by a Landauer-like formula as shown in Refs.^49,50^. Furthermore, the total current flowing from the *i*th ferromagnetic (FM) lead can be expressed as the sum of partial currents corresponding to different tunneling processes:

12 $$\begin{aligned} J_{i}^e= J_{i\uparrow }^e+J_{i\downarrow }^e \end{aligned}$$

with

13 $$\begin{aligned} J_{i\sigma }^e= J_{i\sigma }^{\text{SP}}+J_{i\sigma }^{\text{QP}}+J_{i\sigma }^{\text{DAR}}+J_{i\sigma }^{\text{CAR}}. \end{aligned}$$

Here, $J_{i\sigma }^{\text{SP}}$ is spin-dependent current due to single-particle tunneling from *i*th to *j*th ($j\ne i$) FM lead, obtained as

14 $$\begin{aligned} J_{i\sigma }^{\text{SP}} = \frac{e}{h}\int \text{d}\varepsilon (f_{i}^{-}(\varepsilon ) -f_{j}^{-}(\varepsilon ))T_{ij\sigma }^{\text{SP}}(\varepsilon ). \end{aligned}$$

$J_{i\sigma }^{\text{QP}}$ is quasi-particle current between *i*th FM lead and the superconductor

15 $$\begin{aligned} J_{i\sigma }^{\text{QP}} = \frac{e}{h}\int \text{d}\varepsilon (f_{i}^{-}(\varepsilon ) -f_{3}^{-}(\varepsilon ))T_{i3\sigma }^{\text{QP}}(\varepsilon ) \end{aligned}$$

The next two terms in Eq.(13) correspond to Andreev tunneling processes. Particularly, $J_{i\sigma }^{\text{DAR}}$ denotes current due to direct Andreev reflection, associated with the process in which an electron of a given spin $\uparrow (\downarrow )$ is reflected back as a hole of opposite spin $\downarrow (\uparrow )$ in the same FM lead. The corresponding current is expressed by,

16 $$\begin{aligned} J_{i\sigma }^{\text{DAR}}= \frac{e}{h}\int d\varepsilon (f_{i}^{-}(\varepsilon ) -f_{i}^{+}(\varepsilon ))T_{ii\sigma }^{\text{DAR}}(\varepsilon ). \end{aligned}$$

In turn, the similar processes, however with the hole reflected to the other FM lead than the incoming electron, contribute to cross Andreev current given by

17 $$\begin{aligned} J_{i\sigma }^{\text{CAR}}=\frac{e}{h}\int d\varepsilon (f_{i}^{-}(\varepsilon ) -f_{j}^{+}(\varepsilon ))T_{ij\sigma }^{\text{CAR}}(\varepsilon ) \end{aligned}$$

with $j=2(1)$ for $i=1(2)$. In Eqs. (14-17) the relevant transmission coefficients are defined as, $T_{ij\sigma }^{\text{SP}}(\varepsilon )=G_{\eta \eta }^r[\mathbf {\Gamma }_j{\textbf{G}}^a\mathbf {\Gamma }_i]_{\eta \eta }$,

$T_{i3\sigma }^{\text{QP}}(\varepsilon )=[{\textbf{G}}^r\mathbf {\Gamma }_3{\textbf{G}}^a\mathbf {\Gamma }_1]_{\eta \eta }$ with $\eta =1(3)$ for $\sigma =\uparrow (\downarrow )$, $T_{ii\sigma }^{\text{DAR}}(\varepsilon )=G_{12(34)}^r[\mathbf {\Gamma }_i{\textbf{G}}^a\mathbf {\Gamma }_i]_{21(43)}$, and $T_{ij\sigma }^{\text{CAR}}(\varepsilon )=G_{12(34)}^r[\mathbf {\Gamma }_j{\textbf{G}}^a\mathbf {\Gamma }_i]_{21(43)}$ for $i,j=1,2$ and $\sigma =\uparrow (\downarrow )$.

A positive direction of the particle current (hence negative direction of electron current) is assumed from FM lead to the quantum dot. By virtue of the particle conservation in the closed system, i. e. $\sum _{\sigma }\sum _{i=1,2,3} J_{i\sigma }^e=J_{1}^e+J_{2}^e+J_{3}^e= 0$ , the current flowing out of the superconductor is directly given by the currents out of the remaining electrodes, $J_{3}^e= -J_{1}^e-J_{2}^e$.

Similarly, the heat current, as a result of internal energy change and the Joule heat dissipated due to charge flow, in the *i*th lead can be written as,

18 $$\begin{aligned} J_i^Q=J_{i\uparrow }^Q+J_{i\downarrow }^Q \end{aligned}$$

with

19 $$\begin{aligned} J_{i\sigma }^Q= J_{Qi\sigma }^{\text{SP}}+J_{Qi\sigma }^{\text{AP}}+J_{Qi\sigma }^{\text{DAR}}+J_{Qi\sigma }^{\text{CAR}}. \end{aligned}$$

The relevant contributions to the heat current reads

20a $$\begin{aligned} J_{Qi\sigma }^{\text{SP}}= & \frac{1}{h} \int \text{d}\varepsilon (\varepsilon -\mu _i ) [f_{i}^{-}(\varepsilon )-f_{j}^{-}(\varepsilon )] T_{ij\sigma }^{\text{SP}} (\varepsilon ) \end{aligned}$$

20b $$\begin{aligned} J_{Qi\sigma }^{\text{QP}}= & \frac{1}{h} \int \text{d}\varepsilon (\varepsilon -\mu _i ) [f_{i}^{-}(\varepsilon )-f_{3}^{-}(\varepsilon )] T_{i3\sigma }^{\text{QP}} (\varepsilon ) \end{aligned}$$

20c $$\begin{aligned} J_{Qi\sigma }^{\text{DAR}}= & \frac{-\mu _{i}}{h} \int \text{d}\varepsilon [f_{i}^{-}(\varepsilon )-{f_{i}^{+}}(\varepsilon )] T_{ii\sigma }^{\text{DAR}} (\varepsilon ) \end{aligned}$$

20d $$\begin{aligned} J_{Qi\sigma }^{\text{CAR}}= & \frac{1}{h} \int \text{d}\varepsilon (\varepsilon -\mu _{i})[f_{i}^{-}(\varepsilon ) -f_{j}^{+}(\varepsilon )] T_{ij\sigma }^{\text{CAR}} (\varepsilon ) \end{aligned}$$

with $$j=2(1)$$ for $$i=1(2)$$.

### Thermoelectric coefficients

Let us express the chemical potentials of the FM leads with respect to the superconductor’s chemical potential, i. e. $\mu _{i}=\mu +\delta \mu _i$ for $i=1,2$. We assume the superconducting lead is grounded, i. e. $\mu _3\equiv \mu =0$. Similarly, the temperatures of the FM electrodes are measured with respect to the SC reservoir as $T_{i}=T+\delta T_i$ for $i=1,2$ with $T_3\equiv T$.

Within the linear response approximation, i. e. for $|\delta \mu _i|/k_BT\ll 1$ and $|\delta T_i|/T\ll 1$, we expand particle/charge and heat currents with respect to the driving forces $\delta \mu _i$ and $\delta T_i/T$ up to linear order and obtain;

21 $$\begin{aligned} {\mathbb {J}}={\mathbb {L}}\delta {\mathbb {X}} \end{aligned}$$

particle and thermal current ${\mathbb {J}}=(J_1^n, J_1^Q, J_2^n, J_2^Q)^T$, related to the generalized forces $\delta {\mathbb {X}}=(\delta \mu _1, \delta T_1/T, \delta \mu _2, \delta T_2/T)^T$. Here,

22 $$\begin{aligned} {\mathbb {L}}_\sigma =\frac{1}{h}\int d\varepsilon \left( -\frac{\text {d}f(\varepsilon )}{\text {d}\varepsilon }\right) {\mathbb {T}}_\sigma \end{aligned}$$

with

23 $$\begin{aligned} \begin{aligned} {\mathbb {T}}_\sigma = \begin{pmatrix} {T}_{12\sigma }^{\text{SP}}+{T}_{12\sigma }^{\text{CAR}}+T_{13\sigma }^{\text{QP}}+2T_{11\sigma }^{\text{DAR}} & \varepsilon (T_{12\sigma }^{\text{SP}}+T_{13\sigma }^{\text{QP}}) & -{T}_{12\sigma }^{\text{SP}}+{T}_{12\sigma }^{\text{CAR}} & -\varepsilon T_{12\sigma }^{\text{SP}}\\ \varepsilon (T_{12\sigma }^{\text{SP}}+T_{13\sigma }^{\text{QP}}) & \varepsilon ^{2}(T_{12\sigma }^{\text{SP}}+{T}_{12\sigma }^{\text{CAR}}+T_{13\sigma }^{\text{QP}}) & -\varepsilon T_{12\sigma }^{\text{SP}} & -\varepsilon ^{2}(T_{12\sigma }^{\text{SP}}+{T}_{12\sigma }^{\text{CAR}})\\ -{T}_{21\sigma }^{\text{SP}}+{T}_{21\sigma }^{\text{CAR}} & -\varepsilon T_{21\sigma }^{SP} & {T}_{21\sigma }^{\text{SP}}+{T}_{21\sigma }^{\text{CAR}}+{T}_{23\sigma }^{\text{QP}}+2T_{22\sigma }^{\text{DAR}} & \varepsilon (T_{21\sigma }^{\text{SP}}+T_{23\sigma }^{\text{QP}})\\ -\varepsilon T_{21\sigma }^{\text{SP}} & -\varepsilon ^{2}(T_{21\sigma }^{\text{SP}}+{T}_{21\sigma }^{\text{CAR}}) & \varepsilon (T_{21\sigma }^{\text{SP}}+T_{23\sigma }^{\text{QP}}) & \varepsilon ^{2}(T_{21\sigma }^{\text{SP}}+{T}_{21\sigma }^{\text{CAR}}+T_{23\sigma }^{\text{QP}})\\ \end{pmatrix} \end{aligned} \end{aligned}$$

and ${\mathbb {L}}={\mathbb {L}}_\uparrow +{\mathbb {L}}_\downarrow$. The particle current $J_{i}^n$ is related to the charge current by $J_{i}^e=eJ_{i}^n$. Integrals of the form $\int \text{d}\varepsilon f'(\varepsilon ) \varepsilon {T}_{ij\sigma }^{\text{CAR}}$ vanish as the integrand function is odd, and thus have been omitted. Unlike DAR processes^51,52^, CAR contributes to heat transport in the linear response regime if only $\delta T_1\ne \delta T_2$, which is a key finding of this section.

The local and non-local transport effects, i. e. change in charge (heat) flux out of one lead due to variation in voltage/temperature bias in the same or other reservoir are described by thermoelectric coefficients including electrical conductance ($G_{ij}$), Seebeck coefficient ($S_{ij}$, also called thermopower) and heat conductance ($\kappa _{ij}$). The local thermoelectric coefficients are determined for $i=j=1,2$, whereas non-local ones are given for $i\ne j=1,2$. Unlike a two-terminal device, the definition of these coefficients is not unique for a multi-terminal device. Here, we follow the definition introduced in Ref.^22^ for a three-terminal setup as follows. The generalized electrical conductance, $G_{ij}$, determines the change of current output from *i*th lead due to applied voltage in the *j*th electrode in the absence of temperature bias. More precisely,

24 $$\begin{aligned} G_{ij}= \left( \frac{eJ_{i}^{e}}{\delta \mu _{j}}\right) _{\begin{array}{c} \delta T_{k}=0 \ \forall k \\ \ \ \delta \mu _{k}=0 \ \forall k\ne j \end{array}} \end{aligned}$$

which leads to the following equation for three-terminal system

25 $$\begin{aligned} \left( \begin{array}{cc} G_{11} & G_{12} \\ G_{21} & G_{22} \\ \end{array} \right) = e^2 \left( \begin{array}{cc} L_{11} & L_{13} \\ L_{31} & L_{33} \\ \end{array} \right) . \end{aligned}$$

The thermopower is defined as the voltage developed at a given FM lead by applying temperature bias (measured with respect to SC lead) to the same or different FM electrode under the condition of vanishing charge current out of all FM leads;

26 $$\begin{aligned} S_{ij}= -\left( \frac{\delta \mu _{i}}{e\delta T_j }\right) _{\begin{array}{c} J_k^{e}=0 \ \forall k \\ \ \ \ \ \delta T_{k}=0 \ \forall k\ne j \end{array}}. \end{aligned}$$

In the case of three-terminal system, $S_{ij}$ acquires the form

27 $$\begin{aligned} \begin{aligned} S_{11}&= \frac{1}{eT}\frac{L_{13}L_{32}-L_{12}L_{33}}{(L_{13}L_{31}-L_{11}L_{33})}\\ S_{12}&= \frac{1}{eT}\frac{L_{13}L_{34}-L_{14}L_{33}}{(L_{13}L_{31}-L_{11}L_{33})}\\ S_{22}&= \frac{1}{eT}\frac{L_{31}L_{14}-L_{11}L_{34}}{(L_{13}L_{31}-L_{11}L_{33})}\\ S_{21}&= \frac{1}{eT}\frac{L_{12}L_{31}-L_{11}L_{32}}{(L_{13}L_{31}-L_{11}L_{33})}, \end{aligned} \end{aligned}$$

The generalization of thermal conductance, a quantity that describes the dependence of the heat current on the temperature gradient, assuming no net charge current is flowing through the system, is given by:

28 $$\begin{aligned} \kappa _{ij}=\left( \frac{J_{i}^{Q}}{\delta T_j }\right) _{\begin{array}{c} J_k^{e}=0 \ \forall k \\ \ \ \ \ \ \delta T_{k}=0 \ \ \forall k\ne j \end{array}}. \end{aligned}$$

Direct calculations for three-terminal system by using Eq. (21) leads to;

29 $$\begin{aligned} \begin{aligned} \kappa _{11}&= \frac{1}{T}\frac{(L_{13}L_{32:12})-(L_{12}L_{13:32})-(L_{11}L_{23:23})}{L_{13:31}}\\ \kappa _{12}&= \frac{1}{T}\frac{(L_{24}L_{13:31})+(L_{14}L_{23:13})+(L_{34}L_{12:13})}{L_{13:31}}\\ \kappa _{22}&= \frac{1}{T}\frac{(L_{14}L_{13:43})-(L_{31}L_{14:43})-(L_{11}L_{34:34})}{L_{13:31}}\\ \kappa _{21}&= \frac{1}{T}\frac{(L_{42}L_{13:31})+(L_{41}L_{13:23})+(L_{43}L_{13:12})}{L_{13:31}}, \end{aligned} \end{aligned}$$

with $L_{ij:kl}=L_{ik}L_{jl}-L_{kj}L_{li}$. In general, the heat conductance due to lattice vibration (phonons) should be considered. However, in our treatment, we consider only the electronic contribution to $\kappa _{ij}$, which is plausible since at sufficiently low temperature, the phononic contribution can be negligible compared to the electronic one in a nanoscale system^53–56^.

Along with $G_{ij}$, $S_{ij}$, and $\kappa _{ij}$, we calculated the generalized three-terminal power factor obtained by maximizing linear response output power with respect to $\delta \mu _1$ and $\delta \mu _2$, while keeping $\delta T_1$ and $\delta T_2$ constant. The resulting formula acquires the following form^22^

30 $$\begin{aligned} \text{PF}=\alpha \cos ^2{\theta }+\beta \sin ^2{\theta } \end{aligned}$$

with $\alpha >\beta \ge 0$ denoting the eigenvalues of matrix *M* whose elements depend on the Onsager coefficients (see Supplementary Information for details), while the angle $\theta$ gives the rotation of eigenvectors of *M* in the $\delta T_1/T$, $\delta T_2/T$ plane.

### Spin thermoelectricity

Here, we consider the case where spin voltage induced by spin accumulation in the ferromagnetic electrodes becomes relevant. This can occur due to a sufficiently long spin relaxation time or by applying an external spin bias to the system. In such situations, spin splitting of the ferromagnetic lead’s chemical potential has to be taken into account. Generally, temperature may also be spin-dependent when, in addition to weak spin-flip scattering, inelastic scattering due to electron-electron and electron-phonon interactions is weak, leading to spin heat accumulation^57–60^. However, the energy relaxation time is much shorter than the spin relaxation time and decreases with increasing temperature^57,59,60^. Thus, temperature may be assumed to be independent of spin. We point out that in this section we provide spin-dependent thermoelectric coefficients for three-terminal system which have not been previously reported in the literature.

When spin thermoelectricity becomes relevant, the conditions for determining the thermoelectric coefficients are modified compared to conventional thermoelectricity described in the previous section. Now, the charge and heat currents can be expressed by formulas similar to Eq. (21), but with $\delta \mu _i$ ($i=1,2$) being explicitly spin dependent, i. e. $\delta \mu _i\rightarrow \delta \mu _i^\sigma$, and $L_{ij}$ now includes the derivative of spin dependent Fermi distribution function. More precisely, one can write;

31 $$\begin{aligned} {\mathbb {J}}=\sum _{\sigma }{\mathbb {L}}_\sigma \delta {\mathbb {X}}_\sigma \end{aligned}$$

with

32 $$\begin{aligned} {\mathbb {L}}_\sigma =\frac{1}{h}\int d\varepsilon \left( -\frac{\text {d}f_\sigma (\varepsilon )}{\text {d}\varepsilon }\right) {\mathbb {T}}_\sigma \end{aligned}$$

and $\delta {\mathbb {X}}_\sigma =(\delta \mu _1^\sigma , \delta T_1/T, \delta \mu _2^\sigma , \delta T_2/T)^T$. Spin-dependent difference in chemical potential of $\beta$th lead can be now written

33 $$\begin{aligned} \delta \mu _{i}^{\sigma }=\delta \mu _{i}+{\tilde{\sigma }}\delta \mu _{i}^s \end{aligned}$$

where $\delta \mu _{i}$, as earlier, is chemical potential due to conventional voltage drop, whereas $\delta \mu _{i}^s$ corresponds to spin voltage in *i*th lead due to spin accumulation.

Besides charge conductance , $G_{ij}$, which has the same form as in Eq. (24), one can introduce spin conductance defined as;

34 $$\begin{aligned} G_{ij}^s=\left( \frac{eJ_{i}^{s}}{\delta \mu _j}\right) _{\begin{array}{c} \delta T_k=0 \ \ \forall \ k \\ \delta \mu _k=0 \ \ \forall \ k\ne j \\ \delta \mu _k^s=0 \ \ \forall \ k \end{array}} \end{aligned}$$

with spin current being

35 $$\begin{aligned} J_{i}^{s}=\frac{\hbar }{2e}\sum _{\sigma }{\tilde{\sigma }} J_{i\sigma }^e. \end{aligned}$$

According to the definition (34) one obtains;

36 $$\begin{aligned} \left( \begin{array}{cc} G_{11}^s & G_{12}^s \\ G_{21}^s & G_{22}^s \\ \end{array} \right) = \frac{e\hbar }{2} \sum _{\sigma }{\tilde{\sigma }}\left( \begin{array}{cc} L_{11}^{\sigma } & L_{13}^{\sigma } \\ L_{31}^{\sigma } & L_{33}^{\sigma } \\ \end{array} \right) , \end{aligned}$$

which can be also written using spin-resolved conductance as;

37 $$\begin{aligned} G_{ij}^s=\frac{\hbar }{2e}\sum _{\sigma }{\hat{\sigma }}G_{ij}^\sigma = \frac{\hbar }{2e}(G_{ij}^{\uparrow }-G_{ij}^{\downarrow }), \end{aligned}$$

with $${\tilde{\sigma }}=+ (-)$$ for $$\sigma =\uparrow (\downarrow )$$.

The Seebeck coefficient is now calculated under the condition of vanishing simultaneously both spin current and charge current, or equivalently on the condition of vanishing charge current in each spin channel. As a result, one can define spin-dependent Seebeck coefficient as

38 $$\begin{aligned} S_{ij}^{\sigma }=-\left( \frac{\delta \mu _i^{\sigma }}{e\delta T_j}\right) _{\begin{array}{c} \delta J_k^{\sigma }=0 \ \ \forall \ k,\sigma \\ \delta T_k =0\ \ \forall \ k\ne j \end{array} } \end{aligned}$$

which acquires the form of Eq.(27) substituting $S_{ij}\rightarrow S_{ij}^{\sigma }$ and $L_{\alpha \beta }\rightarrow L_{\alpha \beta }^{\sigma }$ given by Eq. (32). This results in the following expressions for spin Seebeck coefficient;

39 $$\begin{aligned} S_{ij}^{s}=\frac{1}{2}(S_{ij}^{\uparrow }-S_{ij}^{\downarrow }) \end{aligned}$$

and usual charge thermopower

40 $$\begin{aligned} S_{ij}=\frac{1}{2}(S_{ij}^{\uparrow }+S_{ij}^{\downarrow }). \end{aligned}$$

The heat conductance is given by

41 $$\begin{aligned} \kappa _{ij}=\sum _{\sigma }\kappa _{ij}^{\sigma } \end{aligned}$$

with $\kappa _{ij}^{\sigma }$ given by Eq.(29) when substituting $\kappa _{ij}\rightarrow \kappa _{ij}^{\sigma }$ and $L_{ij}\rightarrow L_{ij}^{\sigma }$. Similarly, as in Sec. Thermoelectric coefficients one can introduce three-terminal power factor depending on spin-dependent Onsager’s coefficients. The output power now becomes:

42 $$\begin{aligned} P=-\frac{1}{e}\sum _{\sigma }\sum _{i=1,2}\delta \mu _i^{\sigma }J_{i\sigma }^e \end{aligned}$$

which leads to the following maximum power

43 $$\begin{aligned} P_{max}=\frac{1}{4}\sum _{\sigma }\left( c_{\sigma }\delta T_L^2+2a_{\sigma }\delta T_L\delta T_R+b_{\sigma }\delta T_R^2\right) \end{aligned}$$

with $$c_{\sigma }=G_{11}^{\sigma }(S_{11}^{\sigma })^2+G_{22}^{\sigma }(S_{21}^{\sigma })^2$$$$+(G_{12}^{\sigma }\ +G_{21}^{\sigma })S_{11}^{\sigma }S_{21}^{\sigma },$$ $$b_{\sigma }=G_{11}^{\sigma }(S_{12}^{\sigma })^2+G_{22}^{\sigma }(S_{22}^{\sigma })^2$$$$+(G_{12}^{\sigma } +G_{21}^{\sigma })S_{12}^{\sigma }S_{22}^{\sigma },$$ $$a_{\sigma }=G_{11}^{\sigma }S_{11}^{\sigma }S_{12}^{\sigma }+G_{12}^{\sigma }S_{11}^{\sigma }S_{22}^{\sigma }$$$$+G_{21}^{\sigma }S_{12}^{\sigma }S_{21}^{\sigma }+G_{22}^{\sigma }S_{21}^{\sigma }S_{22}^{\sigma }$$. Equation (43) can be written as

44 $$\begin{aligned} P_{max}=-\frac{1}{4}(\delta T_1,\delta T_2)\sum _{\sigma } M_\sigma \left( \delta T_1,\delta T_2\right) ^T \end{aligned}$$

with

45 $$\begin{aligned} M_\sigma =\left( \begin{array}{cc} c_{\sigma } & a_{\sigma } \\ a_{\sigma } & b_{\sigma } \\ \end{array} \right) . \end{aligned}$$

Diagonalizing Eq. (45) one can introduce power factor similarly as in Sec. Thermoelectric coefficients.

### Heat engine and its efficiency

The prospect of the system to work as a heat engine is parameterized by the efficiency. The general definition of the efficiency of a heat engine is the work done by the system per unit of heat absorbed by it in a given time. In other words, it is the percentage of heat that is transformed into useful work. Within the framework of the model, there is no mechanical work done out of the system but only the work done against the chemical potential due to particle flux driven by a temperature gradient between reservoirs.

Now, the linear response theory is not sufficient to describe the system working as a heat engine. To drive the heat engine and extract power, a finite temperature bias has to be applied at a given lead. In turn, to counteract the thermally induced current one has to apply finite bias voltage. Thus, instead of infinitesimally small quantities $\delta T_i$ and $\delta \mu _i$, one has to introduce finite values $\Delta T_i$ and $\Delta \mu _i$ to describe the system in non-linear regime. Then, charge and heat currents cannot be calculated by Eq. (21) but instead Eq. (12) and Eq. (18) should be used. Again, chemical potentials and temperatures of the FM leads ($i=1,2$) are measured with respect to the superconductor’s chemical potential and temperature, respectively, i. e. $\mu _i=\mu +\Delta \mu _i$ and $T_i=T+\Delta T$ for $i=1,2$.

The work done by the system per unit time is $\sum _i\Delta \mu _iJ_i^n$, and thus, the power generated by the heat engine is,

46 $$\begin{aligned} P=-\frac{1}{e}\sum _i\Delta \mu _iJ_i^e \end{aligned}$$

is equal to heat currents exchanged between the system (QD) and the reservoirs, $\sum _i J_i^Q$. The assumed system is particle and energy conserving. Thus, the heat current *absorbed* by the system is the heat dissipated by the leads i. e. sum of positive heat currents flowing out of the reservoirs, $\sum _{i}^{\prime }J_{i}^{Q}$ with $\sum _{i}^{\prime }$ denoting that the sum is restricted to positive heat currents only. Hence, by following the choice as in Ref.^22^, the efficiency becomes

47 $$\begin{aligned} \eta = \frac{P}{\sum _{i}^{\prime }J_{i}^{Q}} = \frac{1}{e}\frac{-\sum _i\Delta \mu _i J_i^e}{\sum _{i}^{\prime }J_{i}^{Q}}. \end{aligned}$$

The above definition is restricted to the positive output power, $P>0$.

The Carnot efficiency ($\eta _C)$ for the multi-terminal system can not be written merely as a function of the temperature at hottest and coldest leads. Similarly as in two-terminal system it is derived under the condition of none entropy production i. e. $\dot{S} = \sum _i J_{i}^Q/T_i= 0$. Under this constraint, assuming $T_3<T_2<T_1$, Eq. (47) leads to Carnot efficiency^22^:

48a $$\begin{aligned} \eta _{C1}= & 1- \frac{T_{3}}{T_1}+ \frac{J_{2}^Q}{J_{1}^Q}\left( 1-\frac{T_{3}}{T_2}\right) \quad \text{if}\quad J_{1}^Q>0\wedge J_{2}^Q<0 \end{aligned}$$

48b $$\begin{aligned} \eta _{C2}= & 1-\frac{T_{3}}{T_2}+\frac{J_{1}^Q}{J_{2}^Q}\left( 1-\frac{T_{3}}{T_1}\right) \quad \text{if}\quad J_{2}^Q>0\wedge J_{1}^Q<0 \end{aligned}$$

48c $$\begin{aligned} \eta _{C12}= & 1-\frac{1}{J_{1}^Q+J_{2}^Q}\left( \frac{T_{3}}{T_1}J_{1}^Q + \frac{T_{3}}{T_2}J_{2}^Q\right) \quad \text{if}\quad J_{1,2}^Q>0 \end{aligned}$$

Here, for the temperature set $T_3<T_2<T_1$, we consider only the situations when $J_3^Q<0$ and, thus, system can work as heat engine.

## Numerical results and discussions

Considering the linear response regime, we calculate the local and non-local electrical conductance ($G_{ij}$), thermopower ($S_{ij}$), thermal conductance ($\kappa _{ij}$), and the power factor maximized with respect to the applied potential. In the *non-linear* response regime, we calculate and analyze the power output and efficiency of the model device for potential use as a heat engine.

All energy quantities, such as the Coulomb interaction parameter *U*, the quantum dot energy level $\varepsilon _d$, the leads’ temperature $k_{b}T_{i}$, and the QD-lead coupling $\Gamma _{i}$, are given in units of the superconducting gap at zero temperature, $\Delta$. The gap values for typical *s-wave* superconductors range from $(1.5-30) \times 10^{-4}$ eV. Unless stated otherwise, we assume $\Gamma _{1}=0.1\Delta$ and $k_{b}T=0.1\Delta$. To measure asymmetry in the couplings between different pairs of electrodes, we introduce the following parameters: $R= \Gamma _{3}/\Gamma _{1}$ and $A= \Gamma _{2}/\Gamma _{1}$.

### Linear response regime: Conventional thermoelectricity

To analyze the effect of system’s parameters, we individually vary Coulomb repulsion strength *U*, dot’s coupling to a normal *i*th FM (superconducting) lead $\Gamma _i$ ($\Gamma _{3}$), spin polarization factor of *i*th ferromagnetic electrode, $p_i$ Here, we assume that the spin polarization of both ferromagnetic leads are same i. e. $p_1=p_2\equiv p$, if not stated otherwise.

#### Effect of Coulomb repulsion *U*

**Fig. 2:**
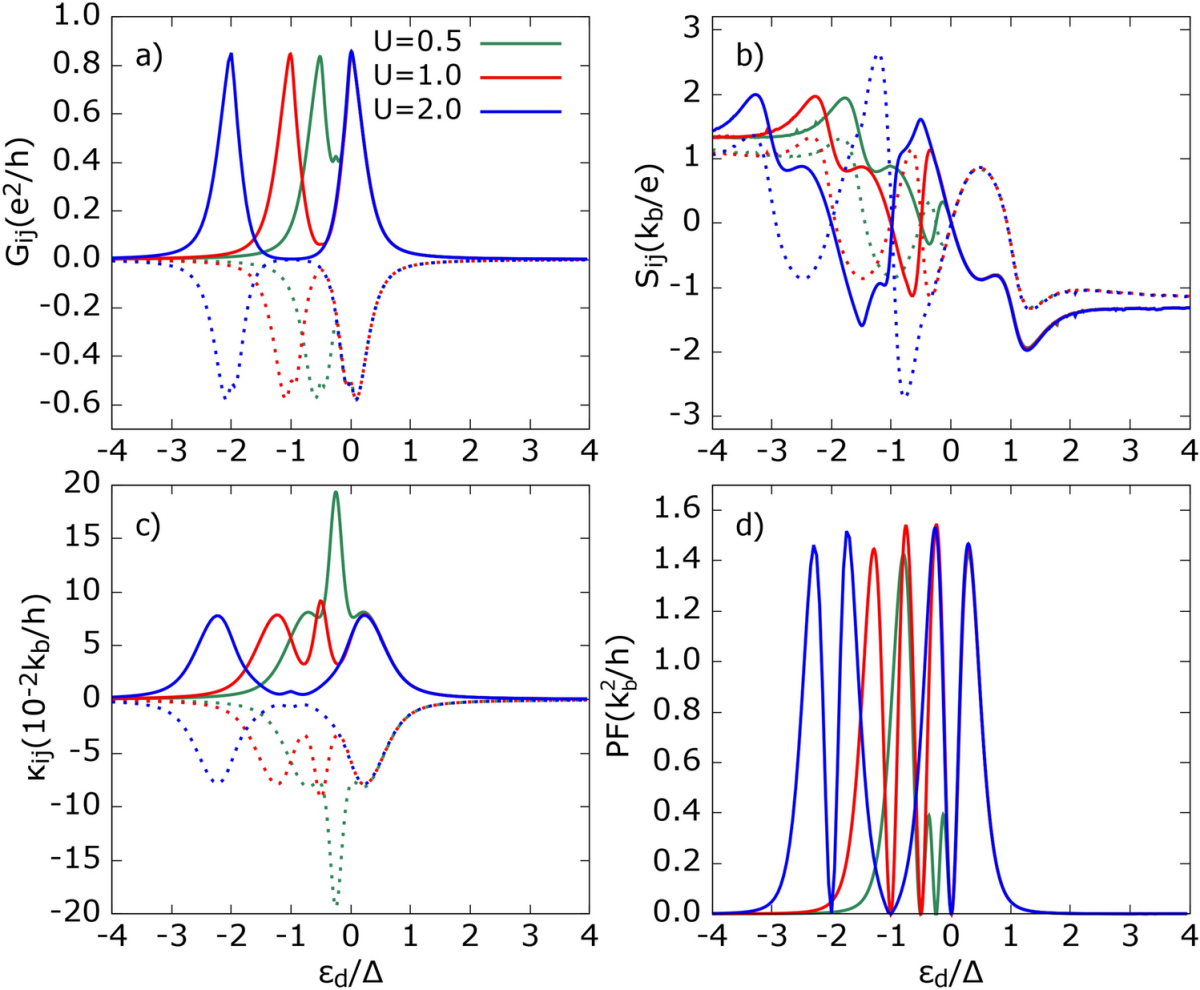
Electrical conductance ($G_{ij}$), thermopower ($S_{ij}$) thermal conductance ($\kappa _{ij}$) and power factor (PF) as a function of dot’s energy level shown for different intra-dot Coulomb potential *U*. Solid lines correspond to local (11) and dotted lines show non-local (12) component of transport coefficients except d). Here, and in all the following figures, we take $e>0$. Other parameters: *p* = 0.5, *R* = 1, $A=$ 1, $\Gamma$ = 0.1$\Delta$, $k_bT$ = 0.1$\Delta$, $\delta \equiv T_1/T_2= -$ 1.

Let us first analyze the effect of intra-dot Coulomb repulsion, *U*. Referring to Fig. 2, we assume the same coupling strength, i. e. $\Gamma _1=\Gamma _2\equiv \Gamma$. Consequently, we show only $X_{11}$ and $X_{12}$, since, in this case, $X_{11}=X_{22}$ and $X_{12}=X_{21}$ for $X=G, S, \kappa$. Note that $G_{11}$ and $G_{12}$ have opposite signs due to the definition (24). This results in a positive sign for CAR and a negative sign for SP tunneling in the non-local conductance $G_{12}$. We are aware that some authors define non-local conductance by multiplying $G_{12}$ by $-1$^61,62^.

As *U* increases, the height of the resonance peaks of $G_{ij}$, shown in Fig. 2a, remains unchanged, but the depth of the valley between the two peaks deepens. The resonance peaks appear at $\varepsilon _{d} \approx 0$ and $\varepsilon _{d} \approx -U$ for both $G_{11}$ and $|G_{12}|$, and the distance between these peaks is proportional to *U*. Analyzing the contributions of different tunneling processes to the charge current, we find that, at a relatively low temperature ($k_bT=0.1\Delta$), SP tunneling processes between FM leads dominate charge transport and thus contribute the most to $G_{ij}$. The individual contributions of tunneling processes to $G_{ij}$ are presented in Fig. S1 of the Supplementary Information. At this temperature, QP tunneling between the SC and FM leads contribute negligibly to the charge current, $J_1^e$, since the thermal energy is insufficient to excite electrons above the superconducting gap. Additionally, there are not enough high-energy electrons in the FM leads to transfer to the SC reservoir. Moreover, the DAR contribution exceeds the CAR contribution at resonance, i. e. for the dot’s energy levels $\varepsilon _d \approx -U, 0$. Interestingly, when considering only DAR and CAR contributions to $G_{11}$ ($|G_{12}|$), the electrical conductance exhibits a small resonance peak at $\varepsilon _d=-U/2$, corresponding to the particle-hole (p-h) symmetry point. However, this feature is obscured in the total conductance due to the partial overlap of conductance peaks. Furthermore, as *U* increases, the height of this small peak at $\varepsilon _d=-U/2$ is suppressed, and it disappears for $U \ge \Delta$. Referring to Eqs. (23) and (24), one can see that only SP and CAR tunneling processes contribute to $G_{12}$. Additionally, the fact that $G_{12}$ is negative indicates that the rate of SP processes contributing to it significantly surpasses that of CAR processes.

Local ($S_{11}$) and non-local ($S_{12}$) thermopower, as a function of the dot’s energy level, calculated for the indicated values of parameter *U*, is presented in Fig. 2b. Recall that $S_{11}$ ($S_{12}$) measures the voltage drop in the FM lead ($i = 1$) induced by the temperature difference $\Delta T_1$ ($\Delta T_2$) between the $i=1$th ($i=2$) FM lead and the SC electrode under open-circuit conditions, i. e. $J_i^e=0$ for $i=1,2$. One notices that for $\varepsilon _d \ge 0$, $S_{ij}$ remains independent of *U*, while for $\varepsilon _d < 0$, the maximum value of thermopower remains roughly the same but occurs at different values of $\varepsilon _d$. The Seebeck coefficient $S_{11}$ vanishes and changes sign at $\varepsilon _d=-U, -U/2, 0$, whereas the thermopower $S_{12}$ also reaches zero at two additional values of $\varepsilon _d$, namely, $\varepsilon _d=-\Delta -U, \Delta$. The vanishing of thermopower $S_{ij}$ indicates that the charge current associated with electrons is exactly compensated by the charge current due to holes.

Interestingly, for $U\le \Delta$, the relation $S_{12}=-S_{11}$ holds for $\varepsilon _d/\Delta \in (-\Delta /2-U,\Delta /2)$, as only SP tunneling processes contribute to heat transfer in the sub-gap region. However, Andreev processes can still influence the Seebeck coefficient indirectly, as they contribute to charge conductance, which appears in the denominator of the thermopower formula [see Eq. (27)]. In contrast, for $U>\Delta$, there exist sub-ranges within the aforementioned $\varepsilon _d$ interval where the equality $S_{12}=-S_{11}$ no longer holds. Additionally, note that negative (positive) values of thermopower $S_{ij}$ indicate that electrons (holes) are the dominant contributors to voltage development. Moreover, increasing *U* enhances $|S_{ij}|$ in the sub-gap region.

Outside the gap region, the equality $S_{12}=-S_{11}$ no longer holds. When the dot’s energy level approaches the superconducting gap edges, i. e. for $\varepsilon _d=-\Delta -U$ and $\varepsilon _d=\Delta$, $S_{ij}$ changes rapidly, and both local and non-local thermopowers acquire the same sign (opposite to that observed in the sub-gap region). A further increase in the dot’s energy level $|\varepsilon _d|$ leads to a gradual decrease in thermopower. However, $S_{ij}$ still exhibits relatively large values due to the strong suppression of electrical conductance, as a relatively large voltage is required to compensate for the thermally induced charge current. Additionally, a small difference arises between $S_{11}$ and $S_{12}$ outside the gap region. This discrepancy can be attributed to the fact that heat transfer via quasiparticle states associated with $S_{11}$ is weighted by both SP and QP charge transfer, whereas for $S_{12}$, it is weighted only by SP processes. More specifically, one can show that the leading terms in thermopower are given by: $S_{11}\propto -\varepsilon T_{13}^{QP}(T_{12}^{SP}+T_{23}^{QP})$ and $S_{12}\propto -\varepsilon T_{23}^{QP}T_{12}^{SP}$ where, for a symmetric system, $T_{13}^{QP}=T_{23}^{QP}$. The integration over $\varepsilon$ has been omitted for clarity.

The heat conductance $\kappa _{ij}$, shown in Fig. 2c, exhibits the symmetry $\kappa _{12}=-\kappa _{11}$ at sufficiently low temperatures. This symmetry arises because, at low temperatures, QP tunneling processes are strongly suppressed, and direct Andreev tunneling processes do not contribute to heat current in a thermally biased system within the linear response regime. Additionally, the heat conductance $|\kappa _{ij}|$ reveals three peaks: two maxima are associated with dot resonances located approximately at $\varepsilon _d=-U, 0$, while a third peak, absent in the electrical conductance, appears at $\varepsilon _d=-U/2$ (the particle-hole symmetry point).

The maximum at $\varepsilon _d=-U$ shifts to lower dot energy levels as *U* increases, whereas the position of the second side peak remains independent of *U*. Moreover, while the height of the side peaks in heat conductance $|\kappa _{ij}|$ does not depend on *U*, the amplitude of the central peak decreases as *U* increases. Notably, the central peak in $|\kappa _{ij}|$ originates from the *bipolar effect*^63^ and is mainly attributed to SP tunneling events. At the particle-hole symmetry point, $\varepsilon _d=-U/2$, the electrical current due to electron flow is completely compensated by the charge current due to holes. However, since electrons and holes flow in the same direction, the resulting heat current is the sum of both contributions, leading to the formation of the central peak. It is worth noting that the peak at $\varepsilon _d=-U/2$ in heat conductance, ascribed to the *bipolar effect*, is absent in a two-terminal FM-QD-SC system^52,64^. However, in such systems, an in-gap feature in heat conductance can appear at sufficiently high temperatures^52^, indicating that only SP tunneling contributes to the *bipolar effect*.

In Fig. 2d, we present the corresponding power factor obtained for the temperature ratio $\delta T_1/\delta T_2=-1$. The height of the power factor exhibits weak dependence on the Coulomb parameter *U*, whereas the intensities of the inner peaks increase with increasing *U*. However, for $U\ge \Delta$, the amplitudes of the inner peaks saturate and surpass those of the outer peaks. This suggests that $U\ge \Delta$ is a necessary condition for achieving good power output in the considered system.

**Fig. 3:**
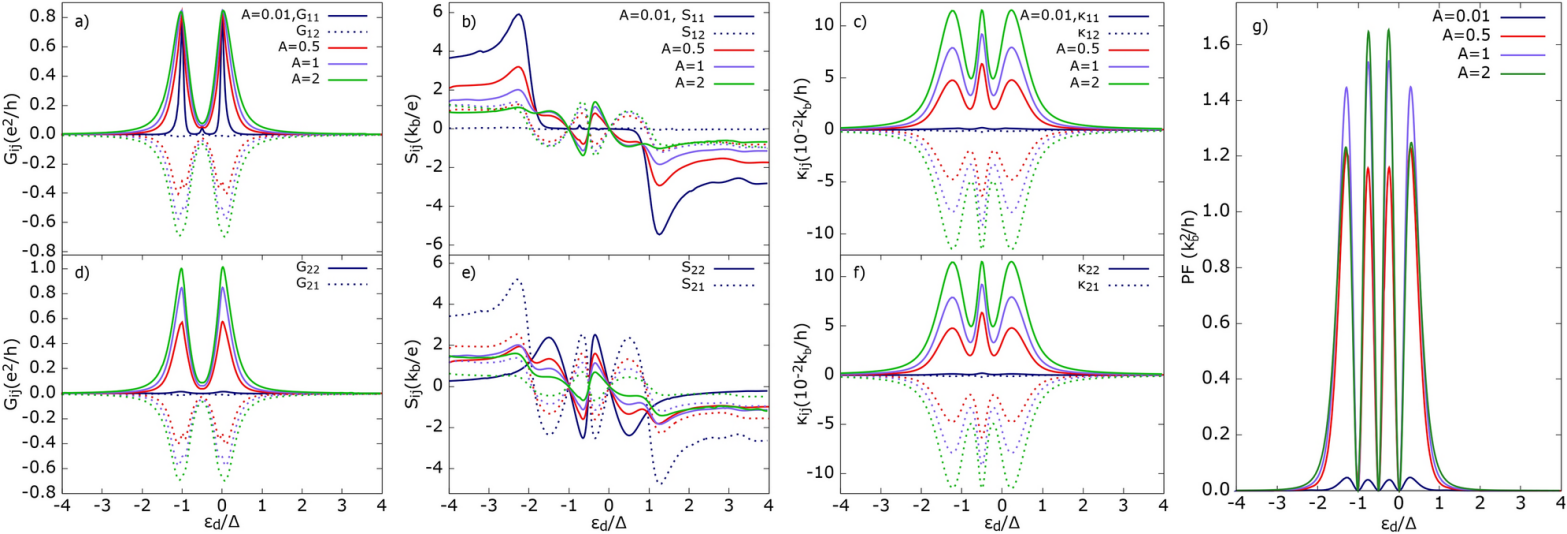
Thermoelectric coefficients: (**a**) and (**d**) electrical conductance, (**b**) and (**e**) thermopower, (**d**) and (**f**) heat conductance, (**g**) power factor, as a function of dot’s energy level calculated for indicated values of parameter A=$\Gamma _{2}/\Gamma _{1}$ denoting asymmetry in dot’s coupling to two FM leads. Solid (dotted) lines correspond to local (non-local) thermoelectric coefficients. Other parameters as in Fig. 2 and $U = \Delta$.

#### Effects of asymmetry in QD’s coupling to two FM leads

Here, we analyze in detail the influence of the second ferromagnetic electrode on the system’s thermoelectric response. Gradually decreasing the quantum dot’s coupling strength to the $\hbox {FM}_2$ lead (until it is fully detached from the dot) allows us to reveal the advantages of the three-terminal system over the two-terminal one. To quantify this effect, we introduce the factor *A*, defined as $A=\Gamma _2/\Gamma _1$, where $\Gamma _2$ is varied while keeping $\Gamma _1$ fixed. In particular, for $A=0$, one of the FM leads is completely detached from the dot.

As the QD couples with different strengths to the two FM leads, the previously mentioned symmetry between various components of the thermoelectric coefficients ceases to exist. In general, the following inequalities hold: $G_{11}\ne G_{22}$ and $S_{11}\ne S_{22}$. However, the relations $G_{12}=G_{21}$ and $\kappa _{11}\approx \kappa _{22}=-\kappa _{12}=-\kappa _{21}$ still hold. Consequently, all components of $S_{ij}$ acquire distinct values.

As shown in Fig. 3a, the peak values of $G_{11}$ do not change significantly with *A*, but the full width at half maximum increases as *A* increases. This indicates that a stronger coupling to the $\hbox {FM}_2$ electrode broadens the resonance in $G_{11}$ due to an enhanced SP tunneling rate. Interestingly, the enhancement of SP tunneling is accompanied by a reduction in DAR processes as *A* increases (see Fig. S2 in the Supplementary Information). Furthermore, the observed increase in $|G_{12}|$ results from contributions of both CAR and SP tunneling processes, as $\Gamma _2$ acts as a scaling factor for the corresponding tunneling amplitudes.

There is a qualitative difference in the behavior of $G_{11}$ and $G_{22}$. Unlike $G_{11}$, the maxima of $G_{22}$ increase significantly with *A*, as shown in Fig. 3d. This can be attributed to the fact that, as the coupling $\Gamma _2$ decreases, not only do SP and CAR processes are weakened, but the DAR tunneling processes between $\hbox {FM}_2$ and the SC are also reduced. In contrast, the DAR tunneling rates between $\hbox {FM}_1$ and the SC show the opposite behavior, as illustrated in Fig. S2 of the Supplementary Information. In turn, $G_{12}$ and $G_{21}$ remain equal for the same value of *A*, which is consistent with the time-reversal symmetry of the Onsager matrix.

Let us now analyze the dependence of the Seebeck coefficients on the parameter *A*, as presented in Fig. 3b and e. First, we explain the behavior of $S_{ij}$ in the sub-gap region. For these dot’s energy levels, it is noticeable that $|S_{11}|$ and $|S_{12}|$ increase with increasing *A*, while $|S_{22}|$ and $|S_{21}|$ show the opposite dependence on *A*. The increase in $|S_{11}|$ and $|S_{12}|$ can result from the fact that, with increasing *A*, heat transfer due to SP processes between the ferromagnetic leads is enhanced. Moreover, this heat transfer is weighted by both local and non-local conductances, $G_{12}$ and $G_{22}$, which also depend on *A*. Specifically, $S_{11(12)}\propto \mp (G_{12}+G_{22})Q_{12}^{\text{SP}}$, where $Q_{12}^{\text{SP}}=\int \text{d}\varepsilon \varepsilon \left( \frac{\text{d}f}{\text{d}\varepsilon }\right) T_{12}^{\text{SP}}(\varepsilon )$. It is evident that all of these quantities become suppressed for small values of *A*, leading to vanishingly small values of $|S_{11}|$ and $|S_{12}|$. Note that $G_{12}$ is negative at resonances; however, the sum $G_{12}+G_{22}$ increases with increasing *A*. More precisely, the numerator of $S_{11(12)}, (G_{12}+G_{22})Q_{12}^{\mathrm{SP}},$ grows much faster than the relevant denominator, $|(G_{12}G_{21}-G_{11}G_{22})|$, as *A* increases.

One can also offer a more physical explanation: AR processes cannot generate a voltage drop due to particle-hole symmetry but can participate in compensating the charge current (reminder: thermopower is defined under this condition). As long as *A* is small and the transport to the $\hbox {FM}_2$ lead is blocked, the only way to transfer charge (between the $\hbox {FM}_1$ and SC electrodes) occurs via AR states, which do not contribute to heat transfer in the linear response regime. Therefore, it is easy to compensate for the thermoelectric current, as $G_{11}$ is finite, by applying a small bias voltage. This results in a small voltage developing in the $\hbox {FM}_1$ lead when a temperature difference, $\delta T_1$ or $\delta T_2$, is applied. Increasing *A* opens the channel to the $\hbox {FM}_2$ lead, and SP processes become more pronounced, leading to a larger voltage drop in $\hbox {FM}_1$ (since a larger voltage bias has to be applied to satisfy the condition of vanishing currents). This, in turn, increases $|S_{11}|$ and $|S_{12}|$. On the other hand, the opposite behavior of $|S_{22}|$ and $|S_{21}|$ can be understood as follows: $S_{22(21)}\propto \mp (G_{21}+G_{11})Q_{12}^{\text{SP}}$, with $G_{11}$ not being strongly dependent on *A* and achieving finite values even for $A\ll 1$, whereas $G_{21}$ vanishes. As *A* increases, $|G_{21}|$ grows, leading to a drop in $G_{21}+G_{11}$ and consequently a decrease in $|S_{22}|$ and $|S_{21}|$. When $A \ll 1$, $G_{12(21)}$ and $G_{22}$ are suppressed, so a large bias voltage must be applied to compensate for even small thermally generated currents, which results in large $|S_{22}|$ and $|S_{21}|$.

Now, we focus on the region outside of the SC gap. One can notice that $|S_{11}|$ and $|S_{21}|$ decrease with increasing *A*, whereas $|S_{12}|$ and $|S_{22}|$ show the opposite behavior. The suppression of $|S_{11}|$ follows from the following facts: outside the gap, heat transfer contributing to $S_{11}$, for small *A* ($A\ll 1$), is mainly driven by quasiparticle tunneling between $\hbox {FM}_1$ and SC leads. However, charge transfer (contributing to conductance and to the denominator of $S_{11}$) also depends on tunneling between ferromagnetic leads (through the tails of the QD level), which decreases with decreasing *A* (see Fig. S2 in the SI). The behavior of $S_{ij}$ above the gap region can be inferred by analyzing approximate formulae, which take the following form:

49a $$\begin{aligned} S_{11}\approx & \frac{1}{T}\frac{Q_{13}^{\text{QP}}}{G_{11}-\frac{G_{12}G_{21}}{G_{22}}} \end{aligned}$$

49b $$\begin{aligned} S_{12}\approx & -\frac{1}{T}\frac{Q_{23}^{\text{QP}}}{|G_{21}|+\frac{G_{11}G_{22}}{|G_{12}|}} \end{aligned}$$

49c $$\begin{aligned} S_{21}\approx & -\frac{1}{T}\left( \frac{Q_{12}^{\text{SP}}}{G_{22}}+\frac{Q_{13}^{\text{QP}}}{|G_{12}|+\frac{G_{11}G_{22}}{|G_{21}|}}\right) \end{aligned}$$

49d $$\begin{aligned} S_{22}\approx & \frac{1}{T}\left( \frac{Q_{12}^{\text{SP}}}{G_{22}}+\frac{Q_{23}^{\text{QP}}}{G_{22}-\frac{G_{12}G_{21}}{G_{11}}}\right) \end{aligned}$$

with $Q_{i3}^{\text{QP}}=\int \text{d}\varepsilon \varepsilon (\text{d}f/\text{d}\varepsilon ) T_{i3}^{\text{QP}}(\varepsilon )$ for $i=1,2$. Equations (49a) and (49b) have been derived by taking $Q_{12}^{\text{SP}} \approx 0$, as thermoelectricity outside the gap is primarily driven by $Q_{i3}^{\text{QP}}$. In contrast, the correct behavior of $S_{21}$ and $S_{22}$ requires considering both terms, i. e. $Q_{i3}^{\text{QP}}$ and $Q_{12}^{\text{SP}}$. The latter term must be included due to the significant variation in $G_{22}$, which appears in the denominator of the first terms in Eqs. (49c) and (49d). Furthermore, the substitution $\pm \frac{G_{21} + G_{11}}{G_{12}G_{21} - G_{11}G_{22}} \rightarrow \mp \frac{1}{G_{22}}$ has been made based on the dependence of $G_{ij}$ on the variation in *A*, which has also been confirmed numerically.

$Q_{13}^{{\text{QP}}}$ (not directly related to the coupling $\Gamma _2$) decreases slowly with increasing *A* due to the competition between QP and SP tunneling processes. In turn, all conductances $|G_{ij}|$ increase with *A*, as described above, but at varying rates. The inequality $G_{11} \ge G_{22} > |G_{12}|$ holds for $A \le 1$. As a result, the *dressed* conductance, ${\tilde{G}}_{11} = G_{11} - \frac{G_{12} G_{21}}{G_{22}}$, which appears in the denominator of Eq. (49a), increases with increasing *A*, further suppressing $|S_{11}|$. The decrease in $|S_{11}|$ can also be explained as follows: For small *A*, $|S_{11}|$ achieves large values, and a large bias voltage must be applied, as ${\tilde{G}}_{11} \approx G_{11}$ is strongly suppressed outside the gap to compensate for the thermocurrent. However, as *A* increases, an additional channel becomes more active, i. e. driven by SP processes, resulting in an increase of ${\tilde{G}}_{11}$, which means a lower bias voltage has to be applied. For small *A*, the temperature difference $\delta T_2$ can only develop a small bias voltage $\delta V_1$ in the $\hbox {FM}_1$ lead because SP processes between $\hbox {FM}_1$ and $\hbox {FM}_2$ are strongly blocked. However, for larger *A*, the SP-driven bias voltage $\delta V_1$ becomes larger, leading to an increase in $|S_{12}|$. The same behavior can be inferred from Eq. (49b). $Q_{23}^{\text{QP}}$ strongly depends on *A* and increases with *A*. Although the *dressed* conductance ${\tilde{G}}_{12}\equiv |G_{21}| + \frac{G_{11} G_{22}}{|G_{12}|}$ also increases with *A*, its growth rate is slower than that of $Q_{23}^{\text{QP}}$, which leads to an increase in $|S_{12}|$. Similarly, the behavior of $S_{21}$ and $S_{22}$ can be explained. Note that in Eq.(49c) and Eq.(49d), an additional factor associated with SP processes is included to ensure the correct behavior for larger values of *A*. Although $S_{21}$ behaves similarly to $S_{11}$, the explanation is slightly more complex. Here, both the numerator and denominator of $S_{21}$ increase with *A*, but the denominator grows faster than the numerator, which leads to the suppression of $|S_{21}|$. In turn, the increase in $|S_{22}|$ with *A* can be attributed to the enhancement of quasiparticle tunneling between $\hbox {FM}_2$ and the SC lead (and also to the increase in SP tunneling rates contributing via the tails of the QD’s level), which drives the heat transfer.

The heat conductance shown in Fig. 3c and f reveals the above-mentioned symmetry, i. e. $\kappa _{11} = -\kappa _{12} = -\kappa _{21} = \kappa _{22}$, regardless of the value of the parameter *A*. In the linear response regime and for sufficiently low temperatures, the heat conductance is mainly driven by SP tunneling processes occurring between the ferromagnetic leads. Thus, it is evident that reducing the coupling to one of the ferromagnetic electrodes results in the suppression of heat conductance, both local and non-local. Note also that the maxima of $|\kappa _{ij}|$ do not coincide exactly with those of the conductances.

Finally, in Fig. 3g, the power factor is presented. The power factor vanishes for dot’s energy levels where the Seebeck coefficients change sign. Interestingly, the intensities of the outer and inner peaks of the PF reveal different behavior. The intensities of the inner peaks rise with increasing *A* and saturate for $A \ge 1$, whereas those of the outer peaks exhibit nonmonotonic behavior: they grow, achieving a maximum for $A = 1$, and then decrease. Regardless of the dot’s energy level, the PF reaches its smallest values for small *A*, i. e. when one of the ferromagnetic leads is almost detached, and the system can be regarded as a two-terminal device. Hence, these results highlight the advantages of a 3-terminal system over a 2-terminal one.

**Fig. 4:**
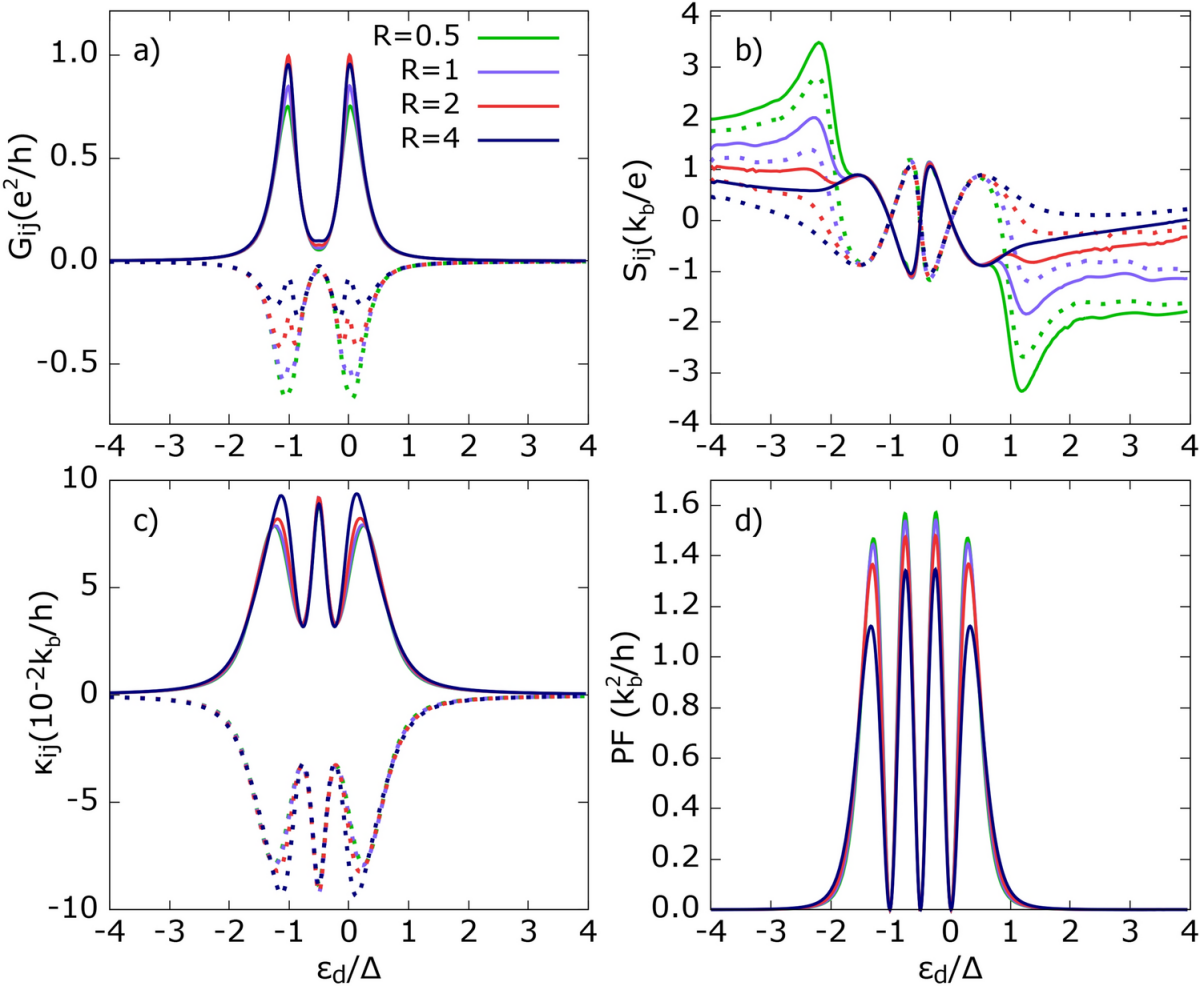
Thermoelectric coefficients: (**a**) electrical conductance, (**b**) thermopower, (**c**) heat conductance, (**d**) power factor as a function of dot’s energy level calculated for indicated values of parameter $R=\Gamma _{3}/\Gamma _{1}$ denoting asymmetry in dot’s coupling to $\hbox {FM}_1$ and SC leads. Solid (dotted) lines correspond to local (non-local) thermoelectric coefficients except (**d**). Other parameters as in Fig. 2 and $U =\Delta$.

#### Effect of QD’s coupling to SC electrode

Now, we analyze the influence of the superconductor on the thermoelectric response of the system. To do this, we gradually detach the SC lead from the QD by decreasing the relevant coupling strength, which is associated with the parameter *R*. In particular, for $R \gg 1$, the SC electrode is much more strongly coupled to the QD than the ferromagnetic leads. Changing the coupling to the SC lead does not break the symmetry between thermoelectric coefficients, i. e. $G_{11} = G_{22}$, $G_{12} = G_{21}$, $\kappa _{11} = \kappa _{22} = -\kappa _{12} = -\kappa _{21}$, and $S_{11} = S_{22}$, $S_{12} = S_{21}$.

One notices that the local electrical conductance $G_{ii}$, shown in Fig. 4a, depends only weakly on changes in *R* and becomes almost independent of *R* for $R > 2$. A drop in local conductance as *R* is reduced originates from the suppression of Andreev processes contributing to the peaks in $G_{ii}$. Simultaneously, the SP contribution increases with decreasing *R*, due to the competition between SP and AR processes, favoring the former, as shown in Fig. S5 in the SI. The dependence of the non-local conductance $G_{i{\bar{i}}}$ on *R* is more pronounced, and $|G_{i{\bar{i}}}|$ decreases with increasing *R*. To understand this behavior, one should recall that only SP and CAR tunneling processes contribute to $G_{i{\bar{i}}}$, and they compete with each other [see e. g. element 13 in Eq. (23)]. Thus, increasing *R* enhances CAR processes while reducing the intensity of SP ones (as shown in Fig. S5 in the Supplementary Information), resulting in a decrease in $|G_{i{\bar{i}}}|$.

The Seebeck coefficient $S_{ij}$, shown in Fig.4b, is independent of variations in *R* when the dot’s energy level is in the range $(-1.5,0.5)\Delta$, as Andreev processes do not contribute to the voltage drop due to particle-hole symmetry. However, outside the SC gap region, both local and non-local thermopower depend significantly on *R*. The drop in $|S_{ij}|$ with increasing *R* can be attributed to an increase in the dressed conductance, e. g. see Eq.(49a), partially enhanced by the increasing contribution of QP, DAR, and CAR tunneling processes (the latter two being provided by the tail of the dot’s energy level). Therefore, a smaller voltage must be applied to compensate for the thermally generated current. For $R \ll 1$, only SP processes contribute due to the tail of the dot’s level, which explains the relatively large values of $|S_{ij}|$.

The heat conductance (Fig.4c) reveals a similar behavior to the electrical conductance $G_{ii}$, i. e. $|\kappa _{ij}|$ slightly increases with increasing *R*. The increase in $|\kappa _{ii}|$ can be attributed to the enhancement of QP tunneling processes, as *R* governs the coupling strength to the SC lead. Finally, increasing *R* leads to an attenuation of the power factor, as shown in Fig.4d.

#### Effect of FM leads’ spin-polarization

**Fig. 5:**
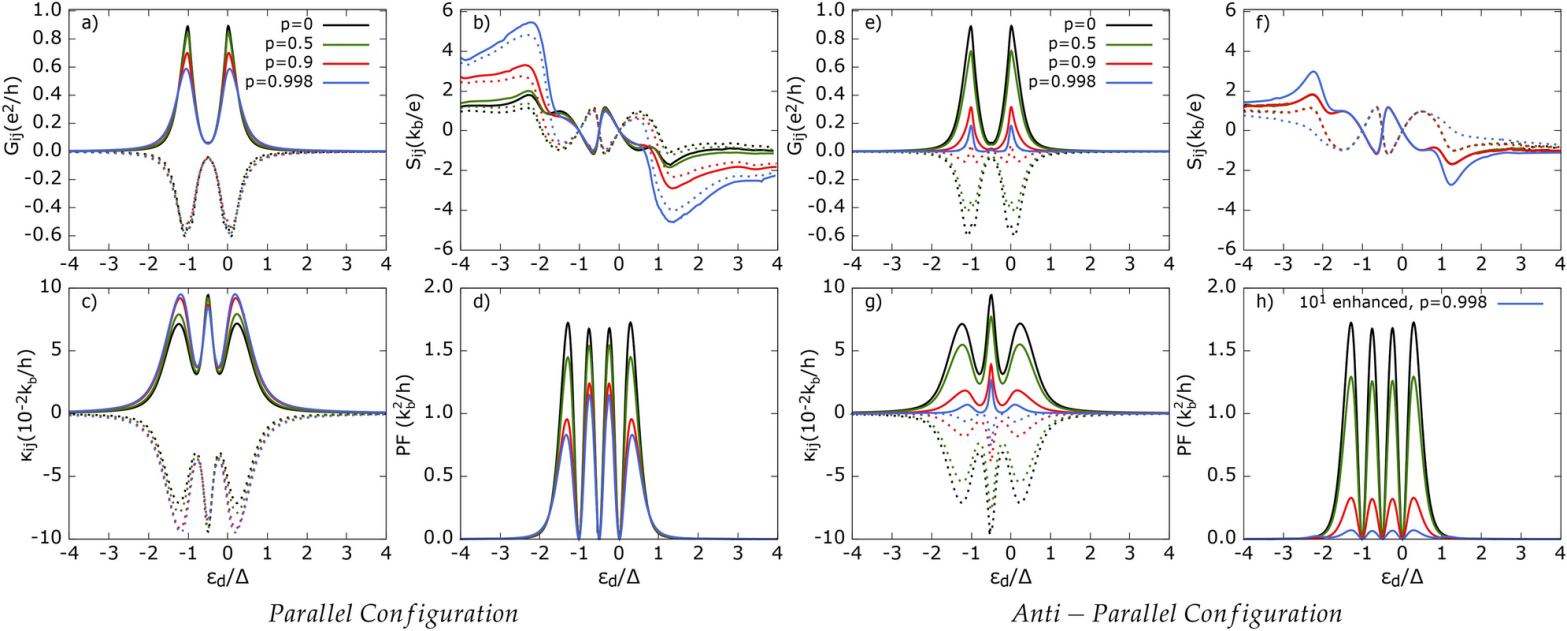
Thermoelectric coefficients: (**a**) and (**e**) electrical conductance, (**b**) and (**f**) thermopower, (**c**) and (**g**) heat conductance, (**d**) and (**h**) power factor as a function of dot’s energy level calculated for indicated values of spin polarization factor *p*. Solid (dotted) lines correspond to local (non-local) thermoelectric coefficients except (**d**) and (**g**). Left (right) pannel shows results for parallel (antiparallel) magnetic configuration. Other parameters as in Fig. 2 and $U =\Delta$.

Exploiting the presence of a ferromagnetic electrode lies in the ability to tune its spin polarization, as demonstrated by various experiments that show multiple ways to achieve this in real materials^65–68^. In a three-terminal system with two ferromagnetic leads, we can vary not only the spin polarization of each ferromagnet but also their relative magnetization alignment. Let us consider the parallel (P) and antiparallel (AP) magnetic configurations, where the magnetic moments of the ferromagnets are aligned in the same or opposite directions, respectively, and analyze how their spin polarization influences the different transport coefficients shown in Fig. 5.

Referring to Fig. 5a and e, we notice that the local electrical conductance maxima decrease with increasing polarization for both P and AP alignments.

However, suppression of the magnitude of the maxima in the latter case is greater than in the former one. Particularly, for high spin polarization, $p\approx 1$, the magnitude of the electrical conductance maxima for P alignment is much greater than that for the AP configuration. In turn, the non-local conductance decreases only for the AP configuration, whereas in the P alignment, it remains nearly unaffected by changes in *p*.

The drop of conductance $G_{ii}$ with increasing *p* in the P alignment can be explained similarly as in Ref.^69^, i. e. due to suppression of AR processes and the gradual narrowing of one of the spin channels between ferromagnetic leads as *p* increases (see also Fig. S3 in the SI). However, in the case of the AP configuration, the reduction of both $G_{ii}$ and $|G_{i{\bar{i}}}|$ is more pronounced and is primarily due to the suppression of SP tunneling processes (see Fig. S4 in the SI). Here, both spin channels are ruled by minority spin carriers, and an increase in *p* leads to the suppression of charge transfer in both channels. Moreover, in the AP alignment, the CAR contribution to both local and non-local conductance increases with *p*. Conversely, for sufficiently large spin polarization, SP and DAR tunneling processes are suppressed. This is evident in $G_{i{\bar{i}}}$, which changes sign for $p\approx 1$, indicating that CAR processes primarily contribute to non-local conductance. Therefore, half-metallic electrodes enable to measure purely CAR conductance.

Note that for half-metallic leads ($p\approx 1$), local and non-local conductances are approximately equal in the AP configuration, as CAR processes predominantly contribute to charge transport. On the other hand, the previously mentioned invariance of $|G_{i{\bar{i}}}|$ for the P alignment arises from the approximately equal decline in both SP and CAR processes with increasing *p*.

As shown in Fig. 5b and f, both local and non-local Seebeck coefficients become insensitive to variations in spin polarization *p* for the AP magnetic configuration in the sub-gap region, whereas a slight change in $|S_{ij}|$ is observed for the P alignment.

For symmetric coupling ($A=1$), $G_{22} = G_{11}$, and one can express the Seebeck coefficient $S_{ij}$ for the gap region for the assumed temperature approximately as:

50 $$\begin{aligned} S_{ij} \approx \pm \frac{1}{T}\frac{Q_{12}^{{\mathrm SP}}}{G_{22}-G_{12}} \end{aligned}$$

with $+( - )$ for $i=j$ ($i\ne j$). Thus, the slight decrease in the peaks of $|S_{ij}|$ in the subgap region for the P alignment can be attributed to a somewhat faster decline in $Q_{12}^{SP}$ compared to $G_{22}-G_{12}$ as *p* increases.

In turn, for the AP alignment, the lack of change in $|S_{ij}|$ in the sub-gap region stems from both $Q_{12}^{SP}$ and the relevant conductance difference drop at the same rate with increasing *p*. Outside the gap region, $|S_{ij}|$ is significantly enhanced with increasing spin polarization, regardless of the magnetic configuration, due to changes in the condition of vanishing charge current under which thermopower is defined.

In the same manner, one can explain the rise of heat conductance $|\kappa _{ij}|$, shown in Fig. 5c, with increasing *p* (as it is also defined at vanishing charge current). As the heat transfer is attenuated with increasing *p* for the AP configuration, the corresponding heat conductance is suppressed, too. Although for $p\approx 1$ the heat conductance $\kappa _{ii}$ achieves relatively small values, it reveals two more peaks corresponding to the dot’s energy levels at $\varepsilon _d=-\Delta -U$ and $\varepsilon _d=\Delta$. The resulting power factor, shown in Fig. 5d and h, diminishes with increasing spin polarization *p*, regardless of the magnetic configuration of the ferromagnetic leads.

## Effect of temperature

**Fig. 6:**
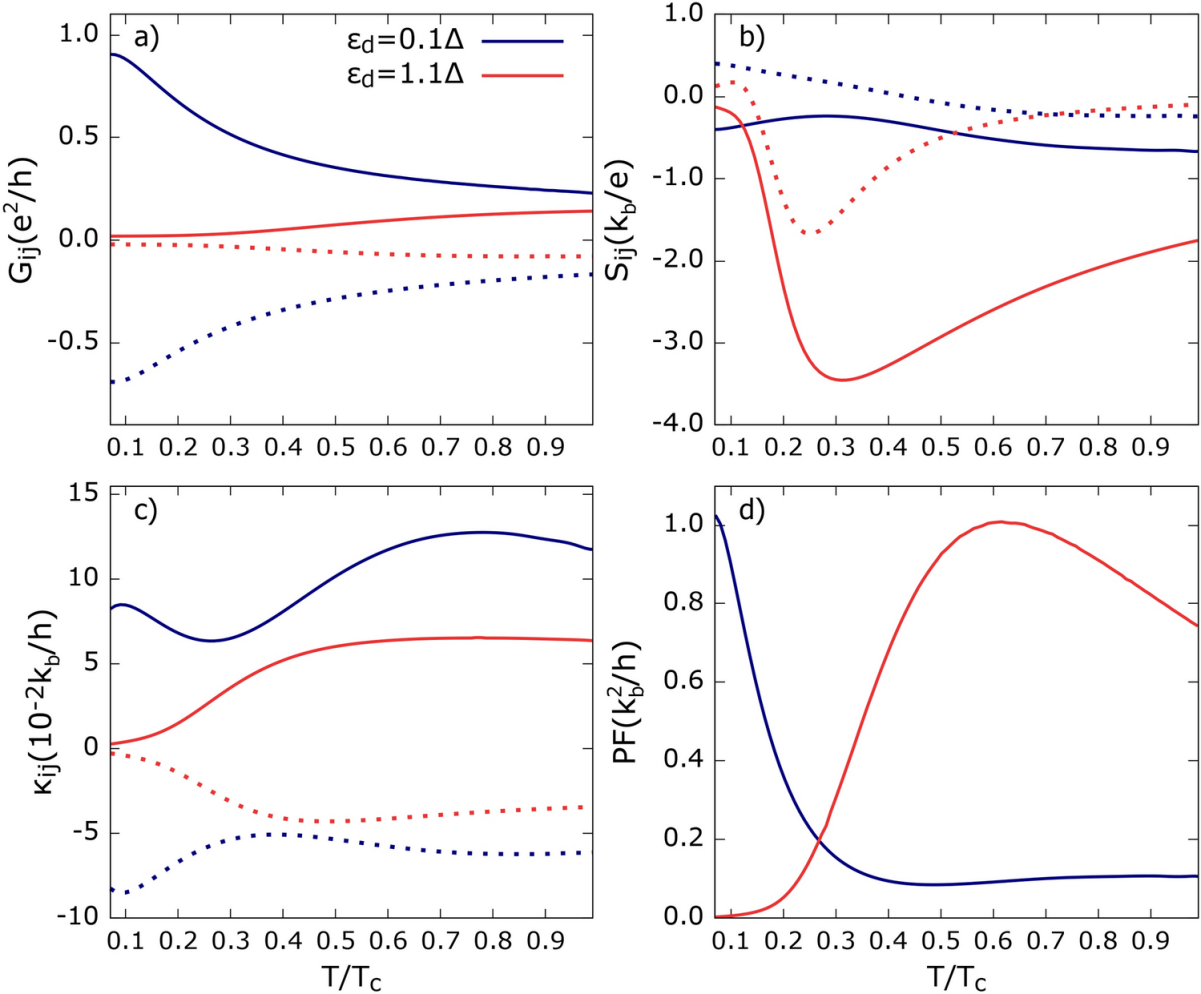
Temperature dependence of transport coefficients: (**a**) electrical conductance, (**b**) thermopower, (**c**) heat conductance, (**d**) power factor calculated for indicated dot’s energy level. Solid (dotted) lines correspond to local (non-local) thermoelectric coefficients except (**d**). Temperature is measured in the units of SC critical temperature $T_c$.

Figure 6 shows the TE coefficients as a function of temperature. The SC gap dependence on *T* is modeled as $\Delta (T)=\Delta (T=0)\sqrt{1-{(T/T_c)}^{3}}$, with the critical temperature being $\Delta (T=0)=1.764k_bT_c$. This formula provides a good fit to the temperature dependence of $\Delta (T)$ obtained with the help of the gap equation^3^. The dot’s energy level is chosen such that it is located inside ($\varepsilon _{d}=0.1\Delta$) or outside ($\varepsilon _{d}=1.1\Delta$) the SC gap. As seen in Fig. 6a, there is a qualitative difference in $G_{ij}$ for $\varepsilon _{d}=0.1\Delta$ and $\varepsilon _{d}=1.1\Delta$. The individual tunneling process contribution analysis shows that all tunneling processes except QP tunneling have a higher contribution to $G_{ij}$ when the dot’s energy is situated deep inside the gap, i. e. for $\varepsilon _{d}=0.1\Delta$, which decreases with temperature. QP processes are activated at higher temperatures, and thus, for $\varepsilon _d$ situated above the SC gap ($\varepsilon _{d}=1.1\Delta$), conductance $|G_{ij}|$ increases with temperature.

Heat conductance, $\kappa _{11}$ and $|\kappa _{12}|$, shown in Fig. 6c, start to differ at sufficiently large temperatures. This can be understood by recalling that QP processes become more pronounced as the temperature increases. Then, they can contribute to heat conductance via the tails of the dot’s level. As QP processes contribute to local and non-local heat conductance to varying degrees, $\kappa _{11}$ and $|\kappa _{12}|$ behave differently with increasing temperature. This is also supported by the fact that the heat conductance for $\varepsilon _{d}=1.1\Delta$, mainly driven by QP processes, is strongly suppressed in the low-temperature regime and reaches significant values at higher temperatures.

Thermopowers $S_{ij}$ shown in Fig. 6b, calculated for a dot’s energy level located deep inside the SC gap, appear to weakly depend on temperature compared to those obtained for $\varepsilon _d>\Delta (T)$. Note that for temperatures very close to $T_c$, the dot’s level is situated outside the SC gap for both the considered cases. Interestingly, $S_{ij}$ for $\varepsilon _{d}=1.1\Delta$, being relatively small at low *T*, attains large values at sufficiently high temperatures. Another interesting feature revealed by non-local thermopower for both cases is that it changes sign at a certain temperature. This feature can be attributed to the increasing role of QP tunneling as temperature rises. Let’s rewrite the expression for $S_{12}$ for symmetric coupling to the FM leads ($A=1$) in the following form:

51 $$\begin{aligned} S_{12}=S_{12}^{\text{SP}}+S_{12}^{\text{QP}} \end{aligned}$$

where

52a $$\begin{aligned} S_{12}^{\text{SP}}\approx & \frac{1}{T}\frac{Q_{12}^{\text{SP}}}{G_{12}-G_{11}}, \end{aligned}$$

52b $$\begin{aligned} S_{12}^{\text{QP}}\approx & \frac{1}{T}\frac{Q_{23}^{\text{QP}}}{G_{12}+\frac{G_{11}^2}{G_{12}}}. \end{aligned}$$

It turns out that $S_{12}^{\text{SP}}$ is non-positive, while $S_{12}^{\text{QP}}$ is non-negative in the considered range of temperatures–see Fig. S6 in SI for details. Regardless of the dot’s energy level, for certain temperatures $S_{12}^{\text{QP}}$ starts to surpass $S_{12}^{\text{SP}}$, and the sign of $S_{12}$ changes. For $\varepsilon _d>\Delta$, the sign change of $S_{12}$ occurs at a lower temperature than for $\varepsilon _d<\Delta$. In the former case, $S_{12}^{\text{QP}}$ starts to contribute to $S_{12}$ at a lower temperature, which results from the monotonic increase of $Q_{23}^{\text{QP}}$ and the suppression of the dressed conductance $G_{12}+\frac{G_{11}^2}{G_{12}}$ for low temperatures.

Finally, the power factor shown in Fig. 6d differs substantially depending on $\varepsilon _d$. Thus, for $\varepsilon _{d}=0.1\Delta$, PF attains relatively large values only at low temperatures, then steeply drops with increasing *T*, reaching a minimum before slightly increasing. On the other hand, PF for $\varepsilon _{d}=1.1\Delta$ attains significant values over a broader temperature range and is therefore preferred. In this case, PF is suppressed in the low-temperature limit and exhibits a maximum at moderate temperatures.

### Linear response regime: spin thermoelectric phenomena

**Fig. 7:**
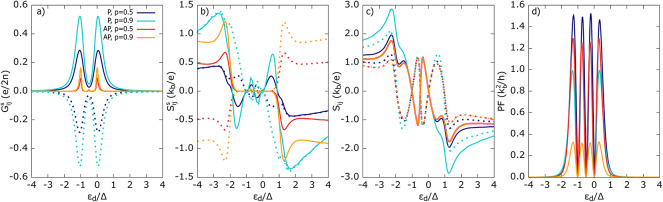
Spin thermoelectric coefficients: (**a**) spin conductance, (**b**) spin thermopower, (**c**) thermopwer (**d**) power factor calculated for parallel and antiparallel magnetic configuration of FM leads. Solid and dotted lines indicate $X_{11}^s$ and $X_{12}^s$ with $X=G, S$ components respectively. Other parameters as in Fig. 2 and $U =\Delta$.

Here, we discuss the spin thermoelectric coefficients in the presence of finite spin accumulation in the FM leads for P and AP magnetic alignment. Regarding Fig. 7a, one can notice that the local and non-local spin conductances are equal (in the sense of absolute value), i. e. $G_{ii}^s = |G_{i{\bar{i}}}^s|$, for sufficiently low temperature. Both $G_{ii}^s$ and $G_{i{\bar{i}}}^s$ in the parallel magnetic configuration are mainly driven by the difference in spin-resolved SP tunneling processes, i. e. with increasing spin polarization factor *p*, more electrons with spin-up ($\uparrow$) orientation tunnel between FM leads than those with spin-down ($\downarrow$) orientation, which results in an enhancement of the spin conductance $|G_{ij}^{s}|$. Note that in the P configuration, both spin contributions to DAR and CAR processes are equal, and thus, they don’t contribute to the spin conductance. Moreover, QP processes are strongly suppressed at the considered temperature.

In contrast, for the AP magnetic alignment, the spin conductance $G_{ij}^{s}$ arises solely from spin-resolved CAR tunneling processes occurring in the gap region. Recall that CAR tunneling requires two electrons with opposite spins coming from different ferromagnetic leads. Moreover, electrons with spin-up in the $\hbox {FM}_1$ lead are majority carriers, whereas those with spin-down are minority carriers. In turn, electrons with spin-down orientation in the $\hbox {FM}_2$ lead are majority carriers, and those with opposite spin are minority carriers. Now, it is clear that processes involving electrons with spin-up orientation coming from $\hbox {FM}_1$ and electrons with spin-down from $\hbox {FM}_2$, contributing to $G_{1j\uparrow }^{\text{CAR}}$, increase with rising spin polarization, whereas CAR processes due to minority electrons coming from both leads, contributing to $G_{1j\downarrow }^{\text{CAR}}$, become suppressed as *p* increases. As a result, the larger the *p*, the greater the difference in spin-resolved conductances, and thus, the larger the spin conductance. This can also be supported by the following formulae: $G_{1j\uparrow }^{\text{CAR}}=\Gamma _1^\uparrow \Gamma _2^\uparrow |G_{12}^r|^2= (1+p)^2\Gamma _1\Gamma _2 |G_{12}^r|^2$, $G_{1j\downarrow }^{\text{CAR}}=\Gamma _1^\downarrow \Gamma _2^\downarrow |G_{34}^r|^2=(1-p^2)\Gamma _1\Gamma _2 |G_{34}^r|^2$, and thus, $G_{1j}^s\propto 2p(1+p)\Gamma _1\Gamma _2 |G_{12}^r|^2$ which clearly prove the above explanation. Note, that Green’s function factors are equal, i. e. $G_{12}^r=G_{34}^r$. Similarly, one can explain the behavior of $G_{2j}^s$. However, here, $G_{2j\uparrow }^{\text{CAR}} = (1 - p^2) \Gamma _1 \Gamma _2 |G_{12}^r|^2$ and $G_{2j\downarrow }^{\text{CAR}} = (1 + p)^2 \Gamma _1 \Gamma _2 |G_{34}^r|^2$, which results in negative $G_{2j}^s \propto -2p(1 + p) \Gamma _1 \Gamma _2 |G_{12}^r|^2$, and $G_{1i}^s = -G_{2j}^s$ for $i, j = 1, 2$. As only CAR processes contribute, local and non-local conductances are equal ($G_{ii}^s = G_{i{\bar{i}}}^s$), not only in the sense of their absolute values. One can conclude that by measuring the spin conductance in the AP configuration, information about pure CAR tunneling can be obtained.

Spin thermopower, shown in Fig. 7b, generally increases with the spin polarization factor *p*, except in the gap region for AP alignment, where $S^s$ vanishes. This feature results from the increasing difference between the two spin components, $S_\uparrow$ and $S_\downarrow$, contributing to spin thermopower. Due to symmetry, one has $S_{11}^s = S_{22}^s$ and $S_{12}^s = S_{21}^s$ for the P alignment and $S_{11}^s =S_{21}^s=-S_{12}^s=-S_{22}^s$ for the AP configuration.

In the gap region for the AP magnetic configuration, both spin channels associated with SP processes contribute equally to the Seebeck effect, i. e. $S_\uparrow = S_\downarrow$, which leads to suppression of spin thermopower. In turn, outside the gap region, $S_{ij}^s$ is finite for both magnetic configurations, and $S_{ij}^s$ for the P alignment has the same sign as the relevant thermopower (cf. Fig. 5b), indicating that $|S_{ij}^\uparrow | > |S_{ij}^\downarrow |$. An approximate formula for $S_{11}^s$ outside the gap region (for P alignment) acquires the following form:

53 $$\begin{aligned} S_{11}^s\approx \frac{1}{2T}\left( \frac{Q_{13\uparrow }^{{\mathrm QP}}}{G_{11\uparrow }-|G_{12\uparrow }|} - \frac{Q_{13\downarrow }^{{\mathrm QP}}}{G_{11\downarrow }-|G_{12\downarrow }|}\right) . \end{aligned}$$

In the P magnetic configuration, $|Q_{13\uparrow }|>|Q_{13\downarrow }|$, while the corresponding denominators (dressed spin-dependent conductances) vary only slightly, $G_{11\uparrow }-|G_{12\uparrow }|\approx G_{11\downarrow }-|G_{12\downarrow }|$, and thus, $|S_{ij}^\uparrow | > |S_{ij}^\downarrow |$. Similar conditions hold for the remaining spin thermopower coefficients. On the other hand, for the AP configuration, one has $S_{11}^s=S_{21}^s=-S_{12}^s=-S_{22}^s$. The opposite sign of, for instance, $S_{12}^s$ compared to $S_{11}^s$ appears due to the fact that $S_{12}^s$ is mainly driven by $Q_{23\sigma }$, which in the AP configuration fulfills the condition $Q_{23\downarrow }>Q_{23\uparrow }$, as in the $\hbox {FM}_2$ lead, electrons with a spin-down orientation are the majority carriers.

For completeness, in Fig.7c, we show the Seebeck coefficient, obtained by using Eq.(40), which follows the same dependence as $S_{ij}$ calculated in the absence of spin accumulation (see Fig. 5b,f) for comparison).

Heat conductance, $\kappa _{ij}$, shown in the Supplementary Information (Fig. S7), for the AP magnetic configuration, follows the electrical conductance behavior, whereas for P alignment, it reveals an opposite dependence on the spin polarization factor. The former feature originates from the suppression of SP tunneling processes with increasing *p*. In turn, the enhancement of $|\kappa _{ij}|$ with increasing *p* for the P alignment can be associated with the suppression of the CAR contribution to the heat current. Moreover, for both magnetic configurations, the following equalities hold: $\kappa _{11}=\kappa _{22}$, $\kappa _{12}=\kappa _{21}$, and for low temperatures, $\kappa _{ii}\approx -\kappa _{i{\bar{i}}}$.

The resulting power factor, calculated in the presence of spin bias, is presented in Fig. 7d for both magnetic configurations of the ferromagnetic leads. Due to the strong suppression of electrical conductance $G_{ij}$ and the rather weak dependence of the thermopower on *p* (especially in the subgap region) for the AP alignment, the corresponding power factor diminishes with increasing spin polarization. Similar behavior can be observed for the P configuration; however, the drop in the PF is not as pronounced as in the AP alignment. This is due to less pronounced decrease in $G_{ij}$ for the P configuration. In conclusion, the power factor follows the trend of its counterpart calculated in the absence of spin bias.

### Non-linear response regime

In this section, we present results on the output power and the corresponding efficiency while the system works as a heat engine. To extract power, one has to apply a bias voltage against the current flowing due to the temperature gradient. Here, we consider two scenarios of biasing the system: (i) a symmetric bias voltage between the FM leads, $V_1=-V_2=V$, (ii) both FM leads are set at the same bias voltage with respect to the grounded SC electrode, $V_2=V_1=V$. The temperature difference, $\Delta T_i$ ($i=1,2$), measured between the *i*-th FM lead and the superconductor, is assumed to be constant. The temperatures of the relevant leads are assumed to fulfill the condition $T_1> T_2 > T_{3}$. The system works as a heat engine when heat is extracted from the hot leads and transferred to the cold one. More specifically, positive heat can flow out of both FM leads or just from one of them, whereas the SC electrode always absorbs heat, i. e. the heat current associated with the SC, $J_3^Q$, has to be negative.

**Fig. 8:**
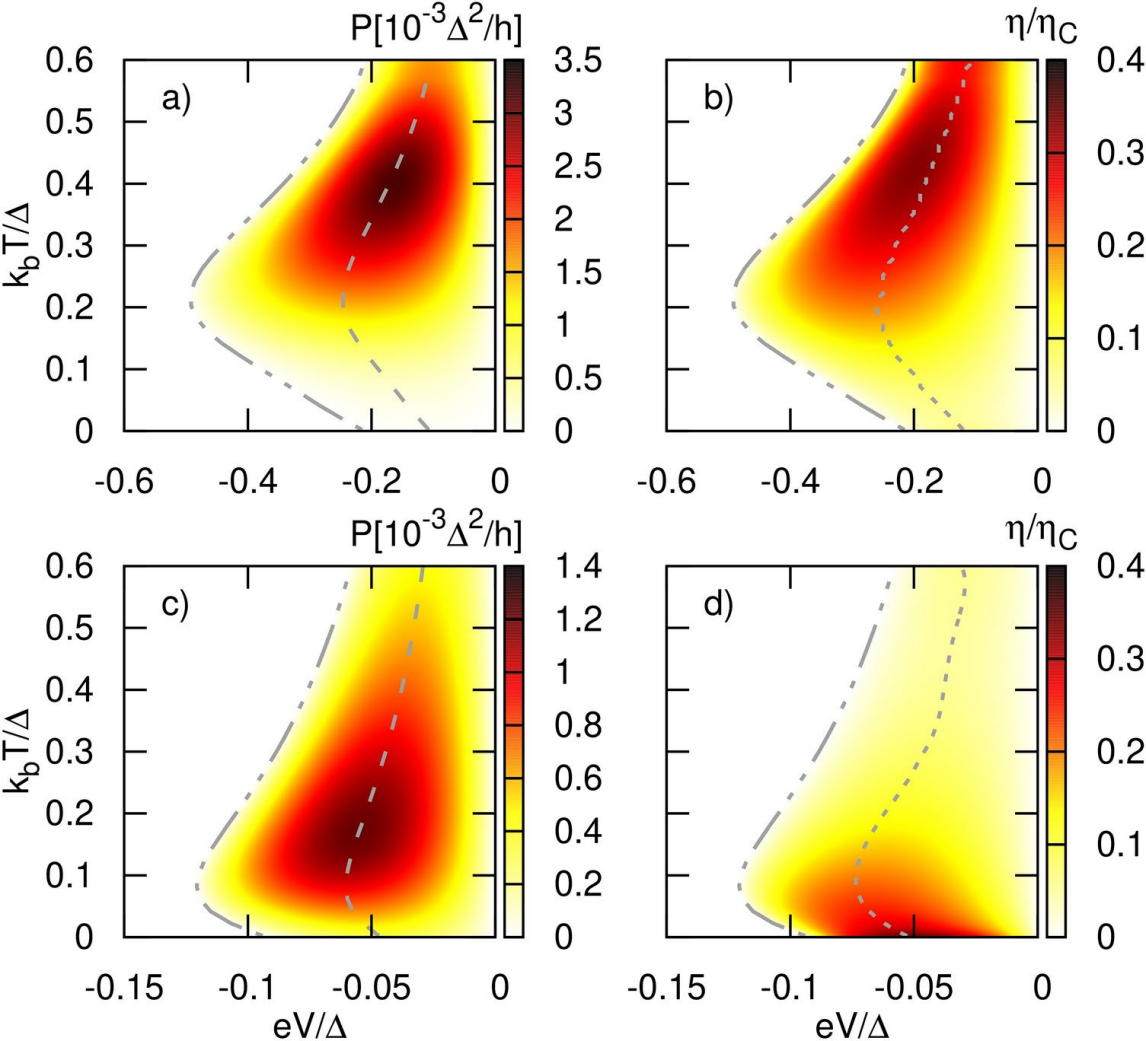
Power (**a**) and (**c**) and relative efficiency (**b**) and (**d**) as a function of bias voltage $eV_1$ and temperature $k_bT$. Upper panel (**a**) and (**b**) corresponds to biasing scenario $V_{1}=V_{2}$, whereas lower panel (**c**) and (**d**) is for $V_{1}=-V_{2}$. The dashed gray line is associated with maximum power (**a**,**c**) and maximum efficiency (**b**,**d**), whereas the dash-dotted gray line indicates the blocking voltage. Other parameters as in Fig. 2 and $U=$ 0, $T_1-T_3=0.2\Delta$, $\Delta T_2-T_3=0.1\Delta$, $\varepsilon _d=\Delta$.

Using the formulae presented in Sec. Heat engine and its efficiency, we numerically calculated the output power and the corresponding efficiency as a function of the applied bias voltage, *eV*, and temperature, $k_bT$. To include temperature dependence of the superconducting gap we introduce, as in Sec. Effect of temperature, an approximate formula for $\Delta (T)$. The corresponding results are displayed in Fig. 8. Note that outside the dash-dotted gray line, indicating the blocking voltage, i. e. the voltage at which the thermally generated current becomes blocked and no output power is generated, the device does not operate as a heat engine.

**Fig. 9:**
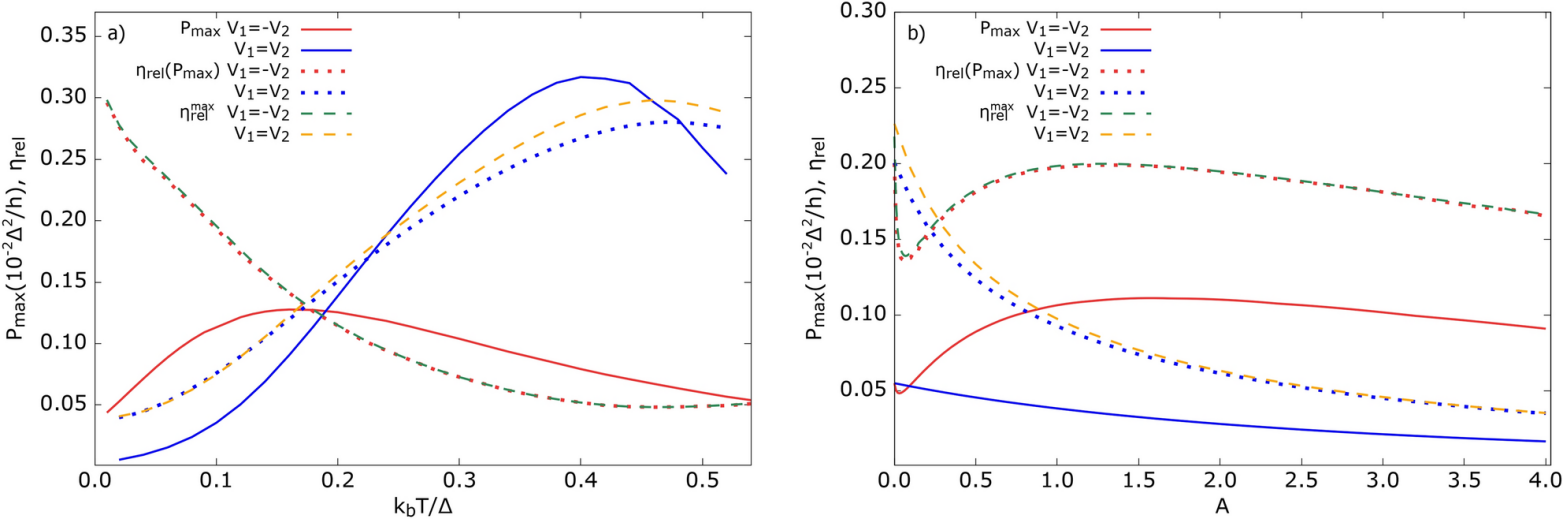
Maximum power ($P_{max}$), efficiency at maximum power [${\eta }_{rel}(P_{max})$] and maximum efficiency ($\eta _{rel}^{max}$) as a function of (**a**) temperature extracted from Fig. 8, (**b**) asymmetry coupling parameter *A*. Here, $\eta _{rel}\equiv \eta /\eta _{C}$.

Generally, a larger power can be extracted for $V_1=V_2$ biasing, but only for relatively high temperatures, $k_bT\approx 0.4\Delta$. For this case, the corresponding efficiency is also relatively high, $\eta /\eta _C\approx 0.3$. Favorably, the highest efficiency approximately coincides with the maximum power. Be aware that, in general, the maximum power does not necessarily follow the maximum efficiency. To support these predictions, Fig. 9a presents results on the maximum power, maximum efficiency, and the efficiency at maximum power as extracted from numerical data. The corresponding efficiency at maximum power fulfills the relation $\eta _{rel}(P_{max})\le \eta _{rel}^{max}$. One can notice that for $V_1=V_2$ biasing, both efficiencies are almost equal up to $k_bT\approx 0.2\Delta$, at which point they begin to drift apart gradually with a further increase in temperature. For symmetrical biasing, $V_1=-V_2$, the highest efficiency is achieved at low temperatures, whereas the largest output power is extracted for $k_bT\approx 0.15\Delta$. In this situation, both $\eta _{rel}(P_{max})$ and $\eta _{rel}^{max}$ are practically the same over the whole temperature range.

To indicate the advantage of a 3-terminal hybrid heat engine over a two-terminal one, in Fig. 9b, we present the operational coefficients as a function of the parameter *A*. Recall that for $A=0$, $\Gamma _2=0$ – the $\hbox {FM}_2$ lead is completely detached from the dot, and the system simplifies to a two-terminal hybrid. It is now clear that the three-terminal system under equal biasing is less effective than the two-terminal one: both the output power and the corresponding efficiency at maximum power are smaller. However, for symmetric biasing, this picture changes considerably in favor of the 3-terminal hybrid. Here, the output power is a non-monotonic function of *A*: it increases, achieving a maximum for a certain value of *A*, and then slowly decreases. However, the power is always greater for $A>0$ within the presented range of *A*, which indicates the advantage of the 3-terminal hybrid over the two-terminal one. Although the largest value of efficiency at maximum power is achieved for $A=0$, it is comparable to that for which the maximum power is maximized. Generally, $\eta _{rel}(P_{max})$ weakly depends on *A* for $A>0.75\Delta$, achieving a maximum for $A\approx 1.25$. In turn, it reveals a minimum in the range $A\in (0,1)$, for $A\approx 0.08$, together with a minimum of $P_{max}$ at $A\approx 0.03$, which suggests that a weakly coupled second normal metal electrode ($\Gamma _2<k_bT$) is insufficient to enhance the performance of the three-terminal hybrid system.

## Summary

We numerically calculated and analyzed the thermoelectric transport properties of a multi-terminal hybrid structure consisting of two ferromagnetic leads and one superconducting electrode connected to a quantum dot. A finite on-dot Coulomb repulsion is assumed within the Hubbard-I approximation. The presence of three terminals demands a non-local response. We explicitly presented the local and non-local thermoelectric coefficients, namely the electrical conductance, thermopower, heat conductance, and the generalized power factor, in the linear response regime. Our focus is to demonstrate the influence of the second ferromagnetic lead on these coefficients. We provided a detailed analysis of the thermoelectric response of the system by varying its different properties, including Coulomb repulsion, the coupling strength of the ferromagnetic and superconducting leads, spin polarization, magnetic configuration and superconductor’s temperature. Furthermore, we show that tuning these parameters can be utilized to optimize the thermoelectric response of the system. The key finding of the introduced theory is that, unlike DAR processes, CAR can contribute to the heat current in the linear response regime.

The presence of second ferromagnetic (FM) electrode enables normal electron tunneling between the two FM leads, which can enhance the thermoelectric response compared to the corresponding two-terminal hybrid system. The obtained power factor clearly demonstrates that greater power can be extracted in a 3-terminal QD-based hybrids than in a two-terminal ones. Apart from that, the obtained results indicate the possibility of achieving an almost pure cross-Andreev current for the antiparallel alignment of the magnetizations of the two FM leads.

Additionally, we introduced the theory of the spin counterparts of thermoelectric coefficients within the linear response limit. We demonstrated that a temperature difference can result in a pronounced spin thermoelectric response of the system, i. e. in the spin-dependent Seebeck effect. We also propose the scheme to measure pure CAR contribution by means of spin conductance. Finally, we presented the potential of a three-terminal hybrid system operating as a heat engine. We showed that the performance of such a heat engine can be further optimized by applying voltage in various configurations, offering certain advantages over two-terminal systems. Our comprehensive analysis of the thermoelectric transport response across various system parameters provides crucial insight to guide future experiments.

## Supplementary Information

Supplementary Information.

## Data Availability

The datasets used and/or analysed during the current study available from the corresponding author on reasonable request.

## References

[1] Benenti, G., Casati, G., Saito, K. & Whitney, R. S. Fundamental aspects of steady-state conversion of heat to work at the nanoscale. *Phys. Rep.* **694**, 1–124. 10.1016/j.physrep.2017.05.008 (2017).

[2] Andreev, A. F. Thermal conductivity of the intermediate state of superconductors. *Zh. Eksperim. i Teor. Fiz.* **46** (1964).

[3] Tinkham, M. *Introduction to Superconductivity* 2nd edn. (Dover Publications, 2004).

[4] Herrmann, L. G. et al. Carbon nanotubes as Cooper-pair beam splitters. *Phys. Rev. Lett.* **104**, 026801. 10.1103/PhysRevLett.104.026801 (2010). 20366615 10.1103/PhysRevLett.104.026801

[5] Braunecker, B., Burset, P. & Levy Yeyati, A. Entanglement detection from conductance measurements in carbon nanotube Cooper pair splitters. *Phys. Rev. Lett.* **111**, 136806. 10.1103/PhysRevLett.111.136806 (2013). 24116805 10.1103/PhysRevLett.111.136806

[6] Tan, Z. B. et al. Thermoelectric current in a graphene Cooper pair splitter. *Nat. Commun.* **12**, 138. 10.1038/s41467-020-20476-7 (2021). 33420055 10.1038/s41467-020-20476-7 PMC7794233

[7] Wang, G. et al. Singlet and triplet Cooper pair splitting in hybrid superconducting nanowires. *Nature*10.1038/s41586-022-05352-2 (2022). 36418399 10.1038/s41586-022-05352-2

[8] Hofstetter, L., Csonka, S., Nygård, J. & Schönenberger, C. Cooper pair splitter realized in a two-quantum-dot y-junction. *Nature* **461**, 960–963 (2009). 19829377 10.1038/nature08432

[9] Schindele, J., Baumgartner, A. & Schönenberger, C. Near-unity Cooper pair splitting efficiency. *Phys. Rev. Lett.* **109**, 157002. 10.1103/PhysRevLett.109.157002 (2012). 23102354 10.1103/PhysRevLett.109.157002

[10] Cao, Z., Fang, T.-F., Li, L. & Luo, H.-G. Thermoelectric-induced unitary Cooper pair splitting efficiency. *Appl. Phys. Lett.* **107**, 212601. 10.1063/1.4936380 (2015).

[11] Kürtössy, O. et al. Parallel InAs nanowires for Cooper pair splitters with Coulomb repulsion. *npj Quant. Mater.* **7**, 88. 10.1038/s41535-022-00497-9 (2022).

[12] Borzenets, I. V. et al. High efficiency CVD graphene-lead (Pb) Cooper pair splitter. *Sci. Rep.* **6**, 23051. 10.1038/srep23051 (2016). 26971450 10.1038/srep23051 PMC4789789

[13] Ginzburg, V. L. Thermoelectric effects in superconductors. *Supercond. Sci. Technol.* **4**, S1. 10.1088/0953-2048/4/1S/001 (1991).

[14] Feng, J.-F., Wu, X.-S. & Jiang, S.-S. Tunneling resonances and Andreev reflection through an interaction quantum dot coupled with two half metals and a superconductor. *J. Appl. Phys.* **99**, 08F713. 10.1063/1.2173625 (2006).

[15] Golubev, D. S. & Zaikin, A. D. Non-local Andreev reflection in superconducting quantum dots. *Phys. Rev. B* **76**, 184510. 10.1103/PhysRevB.76.184510 (2007).

[16] Futterer, D., Governale, M., Pala, M. G. & König, J. Nonlocal Andreev transport through an interacting quantum dot. *Phys. Rev. B* **79**, 054505. 10.1103/PhysRevB.79.054505 (2009).

[17] Büttiker, M. Four-terminal phase-coherent conductance. *Phys. Rev. Lett.* **57**, 1761–1764. 10.1103/PhysRevLett.57.1761 (1986). 10033538 10.1103/PhysRevLett.57.1761

[18] Sánchez, D. & Serra, L. M. C. Thermoelectric transport of mesoscopic conductors coupled to voltage and thermal probes. *Phys. Rev. B* **84**, 201307. 10.1103/PhysRevB.84.201307 (2011).

[19] Machon, P., Eschrig, M. & Belzig, W. Nonlocal thermoelectric effects and nonlocal Onsager relations in a three-terminal proximity-coupled superconductor-ferromagnet device. *Phys. Rev. Lett.* **110**, 047002. 10.1103/PhysRevLett.110.047002 (2013). 25166194 10.1103/PhysRevLett.110.047002

[20] Michałek, G., Urbaniak, M., Bułka, B. R., Domański, T. & Wysokiński, K. I. Local and nonlocal thermopower in three-terminal nanostructures. *Phys. Rev. B* **93**, 235440. 10.1103/PhysRevB.93.235440 (2016).

[21] Wysokiński, K. I. Thermoelectric transport in the three terminal quantum dot. *J. Phys. Condens. Matter* **24**, 335303. 10.1088/0953-8984/24/33/335303 (2012). 22836027 10.1088/0953-8984/24/33/335303

[22] Mazza, F. et al. Thermoelectric efficiency of three-terminal quantum thermal machines. *New J. Phys.* **16**, 085001. 10.1088/1367-2630/16/8/085001 (2014).

[23] Valentini, S., Fazio, R., Giovannetti, V. & Taddei, F. Thermopower of three-terminal topological superconducting systems. *Phys. Rev. B* **91**, 045430. 10.1103/PhysRevB.91.045430 (2015).

[24] Hussein, R. et al. Nonlocal thermoelectricity in a Cooper-pair splitter. *Phys. Rev. B* **99**, 075429. 10.1103/PhysRevB.99.075429 (2019).

[25] Mazza, F. et al. Separation of heat and charge currents for boosted thermoelectric conversion. *Phys. Rev. B*10.1103/PhysRevB.91.245435 (2015).

[26] Josefsson, M., Svilans, A., Linke, H. & Leijnse, M. Optimal power and efficiency of single quantum dot heat engines: Theory and experiment. *Phys. Rev. B* **99**, 235432. 10.1103/PhysRevB.99.235432 (2019).

[27] Tabatabaei, S. M., Sánchez, D., Yeyati, A. L. & Sánchez, R. Nonlocal quantum heat engines made of hybrid superconducting devices. *Phys. Rev. B* **106**, 115419. 10.1103/PhysRevB.106.115419 (2022).

[28] Marchegiani, G., Braggio, A. & Giazotto, F. Superconducting nonlinear thermoelectric heat engine. *Phys. Rev. B* **101**, 214509. 10.1103/PhysRevB.101.214509 (2020).

[29] Sartipi, Z., Hayati, A. & Vahedi, J. Thermoelectric efficiency in three-terminal graphene nano-junctions. *J. Chem. Phys.* **14911**, 114103 (2018). 10.1063/1.5044660 30243286

[30] An, J. H. & Jong, K. H. Optimal thermoelectric induced by Cooper pair splitting. *Phys. B* **654**, 414682. 10.1016/j.physb.2023.414682 (2023).

[31] Barman, A., Halder, S., Varshney, S. K., Dutta, G. & Singha, A. Realistic nonlocal refrigeration engine based on Coulomb-coupled systems. *Phys. Rev. E* **103**, 012131. 10.1103/PhysRevE.103.012131 (2021). 33601520 10.1103/PhysRevE.103.012131

[32] Zhang, Y., Lin, G. & Chen, J. Three-terminal quantum-dot refrigerators. *Phys. Rev. E* **91**, 052118. 10.1103/PhysRevE.91.052118 (2015). 10.1103/PhysRevE.91.052118 26066130

[33] Zhang, Y., Guo, J. & Chen, J. Thermoelectric performance of three-terminal quantum dot refrigerators in two configurations. *Phys. E* **118**, 113874. 10.1016/j.physe.2019.113874 (2020).

[34] Josefsson, M. et al. A quantum-dot heat engine operating close to the thermodynamic efficiency limits. *Nat. Nanotechnol.* **13**, 920–924. 10.1038/s41565-018-0200-5 (2018). 30013221 10.1038/s41565-018-0200-5

[35] Jaliel, G. et al. Experimental realization of a quantum dot energy harvester. *Phys. Rev. Lett.* **123**, 117701. 10.1103/PhysRevLett.123.117701 (2019). 31573223 10.1103/PhysRevLett.123.117701

[36] Svilans, A., Leijnse, M. & Linke, H. Experiments on the thermoelectric properties of quantum dots. *C R Phys.* **17**, 1096–1108. 10.1016/j.crhy.2016.08.002 (2016).

[37] Bauer, G. E. W., Saitoh, E. & van Wees, B. J. Spin caloritronics. *Nat. Mater.* **11**, 391–399. 10.1038/nmat3301 (2012). 22522639 10.1038/nmat3301

[38] Kłobus, W. et al. Entanglement witnessing and quantum cryptography with nonideal ferromagnetic detectors. *Phys. Rev. B* **89**, 125404. 10.1103/PhysRevB.89.125404 (2014).

[39] Trocha, P. & Weymann, I. Spin-resolved Andreev transport through double-quantum-dot Cooper pair splitters. *Phys. Rev. B* **91**, 235424. 10.1103/PhysRevB.91.235424 (2015).

[40] Weymann, I. & Trocha, P. Superconducting proximity effect and zero-bias anomaly in transport through quantum dots weakly attached to ferromagnetic leads. *Phys. Rev. B* **89**, 115305. 10.1103/PhysRevB.89.115305 (2014).

[41] Trocha, P. & Barnaś, J. Spin-polarized Andreev transport influenced by Coulomb repulsion through a two-quantum-dot system. *Phys. Rev. B* **89**, 245418. 10.1103/PhysRevB.89.245418 (2014).

[42] Hofstetter, L. et al. Ferromagnetic proximity effect in a ferromagnet-quantum-dot-superconductor device. *Phys. Rev. Lett.* **104**, 246804. 10.1103/PhysRevLett.104.246804 (2010). 20867324 10.1103/PhysRevLett.104.246804

[43] Hamaya, K. et al. Spin transport through a single self-assembled inas quantum dot with ferromagnetic leads. *Appl. Phys. Lett.* **90**, 053108. 10.1063/1.2435957 (2007).

[44] Hamaya, K. et al. Electric-field control of tunneling magnetoresistance effect in a ni/inas/ni quantum-dot spin valve. *Appl. Phys. Lett.* **91**, 022107. 10.1063/1.2759264 (2007).

[45] Hamaya, K. *et al.* Kondo effect in a semiconductor quantum dot coupled to ferromagnetic electrodes. *Applied Physics Letters* **91**, 232105, 10.1063/1.2820445 (2007). https://pubs.aip.org/aip/apl/article-pdf/doi/10.1063/1.2820445/14385246/232105_1_online.pdf.

[46] Nambu, Y. Quasi-particles and gauge invariance in the theory of superconductivity. *Phys. Rev.* **117**, 648–663. 10.1103/PhysRev.117.648 (1960).

[47] Zubarev, D. N. Double-time Green functions in statistical physics. *Soviet Physics Uspekhi* **3**, 320–345. 10.1070/pu1960v003n03abeh003275 (1960).

[48] Trocha, P. & Barnaś, J. Quantum interference and Coulomb correlation effects in spin-polarized transport through two coupled quantum dots. *Phys. Rev. B* **76**, 165432. 10.1103/PhysRevB.76.165432 (2007).

[49] Meir, Y. & Wingreen, N. S. Landauer formula for the current through an interacting electron region. *Phys. Rev. Lett.* **68**, 2512–2515. 10.1103/PhysRevLett.68.2512 (1992). 10045416 10.1103/PhysRevLett.68.2512

[50] Zeng, Z. Y., Li, B. & Claro, F. Electronic transport in hybrid mesoscopic structures: A nonequilibrium Green function approach. *Phys. Rev. B* **68**, 115319. 10.1103/PhysRevB.68.115319 (2003).

[51] Hwang, S.-Y., López, R. & Sánchez, D. Large thermoelectric power and figure of merit in a ferromagnetic-quantum dot-superconducting device. *Phys. Rev. B* **94**, 054506. 10.1103/PhysRevB.94.054506 (2016).

[52] Trocha, P. & Barnaś, J. Spin-dependent thermoelectric phenomena in a quantum dot attached to ferromagnetic and superconducting electrodes. *Phys. Rev. B* **95**, 165439. 10.1103/PhysRevB.95.165439 (2017).

[53] Hochbaum, A. I. et al. Enhanced thermoelectric performance of rough silicon nanowires. *Nature* **451**, 163–167. 10.1038/nature06381 (2008). 18185582 10.1038/nature06381

[54] Boukai, A. I. et al. Silicon nanowires as efficient thermoelectric materials. *Nature* **451**, 168–171. 10.1038/nature06458 (2008). 18185583 10.1038/nature06458

[55] Markussen, T., Jauho, A.-P. & Brandbyge, M. Surface-decorated silicon nanowires: A route to high- thermoelectrics. *Phys. Rev. Lett.* **103**, 055502. 10.1103/PhysRevLett.103.055502 (2009). 19792512 10.1103/PhysRevLett.103.055502

[56] Kuo, D.M.-T. & Chang, Y.-C. Thermoelectric and thermal rectification properties of quantum dot junctions. *Phys. Rev. B* **81**, 205321. 10.1103/PhysRevB.81.205321 (2010).

[57] Hatami, M., Bauer, G. E. W., Zhang, Q. & Kelly, P. J. Thermal spin-transfer torque in magnetoelectronic devices. *Phys. Rev. Lett.* **99**, 066603. 10.1103/PhysRevLett.99.066603 (2007). 17930848 10.1103/PhysRevLett.99.066603

[58] Hatami, M., Bauer, G. E. W., Zhang, Q. & Kelly, P. J. Thermoelectric effects in magnetic nanostructures. *Phys. Rev. B* **79**, 174426. 10.1103/PhysRevB.79.174426 (2009).

[59] Heikkilä, T. T., Hatami, M. & Bauer, G. E. W. Spin heat accumulation and its relaxation in spin valves. *Phys. Rev. B* **81**, 100408. 10.1103/PhysRevB.81.100408 (2010).

[60] Dejene, F. K., Flipse, J., Bauer, G. E. W. & van Wees, B. J. Spin heat accumulation and spin-dependent temperatures in nanopillar spin valves. *Nat. Phys.* **9**, 636–639. 10.1038/nphys2743 (2013).

[61] Michałek, G., Bułka, B. R., Domański, T. & Wysokiński, K. I. Interplay between direct and crossed Andreev reflections in hybrid nanostructures. *Phys. Rev. B* **88**, 155425. 10.1103/PhysRevB.88.155425 (2013).

[62] Futterer, D., Governale, M. & König, J. Generation of pure spin currents by superconducting proximity effect in quantum dots. *Europhys. Lett.* **91**, 47004. 10.1209/0295-5075/91/47004 (2010).

[63] Trocha, P. & Barnaś, J. Large enhancement of thermoelectric effects in a double quantum dot system due to interference and Coulomb correlation phenomena. *Phys. Rev. B* **85**, 085408. 10.1103/PhysRevB.85.085408 (2012).

[64] Verma, S. & Singh, A. Non-equilibrium thermoelectric transport across normal metal-quantum dot-superconductor hybrid system within the Coulomb blockade regime. *J. Phys.: Condens. Matter* **34**, 155601. 10.1088/1361-648X/ac4ced (2022). 10.1088/1361-648X/ac4ced 35045407

[65] Korenev, V. L. et al. Dynamic spin polarization by orientation-dependent separation in a ferromagnet-semiconductor hybrid. *Nat. Commun.* **3**, 959. 10.1038/ncomms1957 (2012). 22805566 10.1038/ncomms1957

[66] Qiu, Z. J., Zhang, S.-L. & Liu, R. Tuning of spin polarization in ferromagnetic resonant tunneling diode by varying doping. *Appl. Phys. Lett.* **92**, 242110. 10.1063/1.2949684 (2008).

[67] Wang, L. et al. Composition controlled spin polarization in : Electronic, magnetic, and thermodynamic properties. *Phys. Rev. B* **73**, 144402. 10.1103/PhysRevB.73.144402 (2006).

[68] Atodiresei, N. et al. Design of the local spin polarization at the organic-ferromagnetic interface. *Phys. Rev. Lett.* **105**, 066601. 10.1103/PhysRevLett.105.066601 (2010). 20867994 10.1103/PhysRevLett.105.066601

[69] Sonar, V. & Trocha, P. Non-local thermoelectric transport in multi-terminal quantum dot hybrid system. *J. Magn. Magn. Mater.* **593**, 171745. 10.1016/j.jmmm.2024.171745 (2024).

