# Supplementary material for: Spin dependent thermoelectric transport in a multiterminal quantum dot hybrid including a superconductor and ferromagnets
Source: Sci Rep. 2025 Apr 25;15:14509. doi: 10.1038/s41598-025-94991-2 (PMC12032268; doi:10.1038/s41598-025-94991-2)
Supplement: Supplementary file 1 — Supplementary Information. [file 41598_2025_94991_MOESM1_ESM.pdf]

Spin dependent thermoelectric transport in a multiterminal quantum dot hybrid including a superconductor and ferromagnets

Vrishali Sonar[1,*] **and Piotr Trocha**[1,†]

[1]Institute of Spintronics and Quantum Information, Faculty of Physics and Astronomy, Adam Mickiewicz University, Poznań, 61-614, Poland
[†]ptrocha@amu.edu.pl
[*]vrison@amu.edu.pl

Green's functions

The retarded Green's function 4×4 matrix represented in Nambu space using Zubarev notation is given as,

$$\mathbf{G}^r = \left\langle\left\langle \begin{pmatrix} d_\uparrow & d_\downarrow^\dagger & d_\downarrow & d_\uparrow^\dagger \end{pmatrix}^T \mid \begin{pmatrix} d_\uparrow^\dagger & d_\downarrow & d_\downarrow^\dagger & d_\uparrow \end{pmatrix} \right\rangle\right\rangle. \tag{1}$$

Due to Hamiltonian symmetry only eight elements of the matrix are finite and $\mathbf{G}^r$ is block-diagonal i. e. the only non-zero elements read, G^r_{ij} for $i,j = 1,2$ and G^r_{ij} for $i,j = 3,4$. Using equation of motion technique we obtain $\mathbf{G}^r$ within Hubbard I approximation. The explicit form of these elements are as follow,

$$G^r_{11}(\varepsilon) = \frac{\left(1 + \frac{Un_\downarrow}{\varepsilon-\varepsilon_d-U}\right)}{\varepsilon - \varepsilon_d - \Sigma^r_{11}\left(1 + \frac{Un_\downarrow}{\varepsilon-\varepsilon_d-U}\right) - P_1\left(\Sigma^r_{12}(1 + \frac{Un_\downarrow}{\varepsilon-\varepsilon_d-U}\right)}, \tag{2a}$$

$$G^r_{22}(\varepsilon) = \frac{\left(1 - \frac{Un_\uparrow}{\varepsilon+\varepsilon_d+U}\right)}{\varepsilon + \varepsilon_d - \Sigma^r_{22}\left(1 - \frac{Un_\uparrow}{\varepsilon+\varepsilon_d+U}\right) - Q_1\left(\Sigma^r_{21}(1 - \frac{Un_\uparrow}{\varepsilon+\varepsilon_d+U}\right)}, \tag{2b}$$

$$G^r_{12}(\varepsilon) = \frac{Q_1\left(1 - \frac{Un_\uparrow}{\varepsilon+\varepsilon_d+U}\right)}{\varepsilon + \varepsilon_d - \Sigma^r_{22}\left(1 - \frac{Un_\uparrow}{\varepsilon+\varepsilon_d+U}\right) - Q_1\Sigma^r_{21}\left(1 - \frac{Un_\uparrow}{\varepsilon+\varepsilon_d+U}\right)}, \tag{2c}$$

$$G^r_{21}(\varepsilon) = \frac{P_1\left(1 + \frac{Un_\downarrow}{\varepsilon-\varepsilon_d-U}\right)}{\varepsilon - \varepsilon_d - \Sigma^r_{11}\left(1 + \frac{Un_\downarrow}{\varepsilon-\varepsilon_d-U}\right) - P_1\Sigma^r_{12}\left(1 + \frac{Un_\downarrow}{\varepsilon-\varepsilon_d-U}\right)} \tag{2d}$$

where Σ^r_{ij} is relevant element of retarded self-energy, $\Sigma^r = \Sigma^r_1 + \Sigma^r_2 + \Sigma^r_3$, introduced in the main text, and

$$P_1 = \frac{\Sigma^r_{21}(\varepsilon + \varepsilon_d + U - Un_\uparrow)}{(\varepsilon + \varepsilon_d)(\varepsilon + \varepsilon_d + U) - \Sigma^r_{22}(\varepsilon + \varepsilon_d + U - Un_\uparrow)}, \tag{3a}$$

$$Q_1 = \frac{\Sigma^r_{12}(\varepsilon - \varepsilon_d - U + Un_\downarrow)}{(\varepsilon - \varepsilon_d)(\varepsilon - \varepsilon_d - U) - \Sigma^r_{11}(\varepsilon - \varepsilon_d - U + Un_\downarrow)}. \tag{3b}$$

Similarly, G^r_{33}, G^r_{34}, G^r_{43}, G^r_{44} can be obtained by replacing $\uparrow (\downarrow)$ by $\downarrow (\uparrow)$, and $P_1 \to P_2$, $Q_1 \to Q_2$ with

$$P_2 = \frac{\Sigma^r_{43}(\varepsilon + \varepsilon_d + U - Un_\downarrow)}{(\varepsilon + \varepsilon_d)(\varepsilon + \varepsilon_d + U) - \Sigma^r_{44}(\varepsilon + \varepsilon_d + U - Un_\downarrow)}, \tag{4a}$$

$$Q_2 = \frac{\Sigma^r_{34}(\varepsilon - \varepsilon_d - U + Un_\uparrow)}{(\varepsilon-\varepsilon_d)(\varepsilon-\varepsilon_d-U) - \Sigma^r_{33}(\varepsilon-\varepsilon_d-U+Un_\uparrow)}. \tag{4b}$$

In the above equations, the mean value $\langle d_\downarrow d_\uparrow \rangle$ has been omitted as except for very low temperature, it is vanishingly small[1,2]. The expectation value of dot's occupation number for both spin orientations is calculated self-consistently using the identity,

$$\langle n_{\uparrow(\downarrow)} \rangle = -i \int \frac{d\varepsilon}{2\pi} G^<_{11(33)}(\varepsilon). \tag{5}$$

Three-terminal power factor

The maximum power for given temperature differences δT_1 and δT_2 can be calculated by the maximization of the output power with respect to $\delta\mu_1$ and $\delta\mu_2$ (keeping δT_1 and δT_2 constant). The power generated by system is given as

$$P = -(J^n_1 \delta\mu_1 + J^n_2 \delta\mu_2) \tag{6}$$

Then, with the help of Eq. (20) one finds maximum power P_{max} which acquires the form:

$$P_{max} = \frac{1}{4}\mathbb{T}^T \mathbb{M} \mathbb{T} \tag{7}$$

with $\mathbb{T} = (\delta T_1, \delta T_2)^T$ and

$$\mathbb{M} = \begin{pmatrix} c & a \\ a & b \end{pmatrix}. \tag{8}$$

Elements of the matrix $\mathbb{M}$ are expressed by Onsager's coefficients via the following relations

$$\begin{aligned} a &= G_{11}S_{11}S_{12} + G_{12}S_{11}S_{22} + G_{21}S_{12}S_{21} + G_{22}S_{21}S_{22}, \\ b &= G_{11}S^2_{12} + G_{22}S^2_{22} + (G_{12}+G_{21})S_{12}S_{22}, \\ c &= G_{11}S^2_{11} + G_{22}S^2_{21} + (G_{12}+G_{21})S_{11}S_{21}. \end{aligned} \tag{9}$$

Eq. (7) can be written in the bra-ket notation as

$$P_{max} = \frac{1}{4}\langle \mathbb{T}|\mathbb{M}|\mathbb{T}\rangle \tag{10}$$

where we introduced $|\mathbb{T}\rangle \equiv \mathbb{T}$. Utilizing completeness relation, $|\alpha\rangle\langle\alpha| + |\beta\rangle\langle\beta| = \mathbb{I}$, one obtains

$$P_{max} = \frac{1}{4}\left(\alpha|\langle\mathbb{T}|\rangle|^2 + \beta|\langle\mathbb{T}|\beta\rangle|^2\right) \tag{11}$$

with $\langle\mathbb{T}|\alpha\rangle = \delta T_1\alpha_1 + \delta T_2\alpha_2$ and $\langle\mathbb{T}|\beta\rangle = \delta T_1\beta_1 + \delta T_2\beta_2$. Here, $|\alpha\rangle = (\alpha_1,\alpha_2)^T$ and $|\beta\rangle = (\beta_1,\beta_2)^T$ are eigenvectors corresponding to eigenvalues α and β of $\mathbb{M}$ and are given by:

$$\begin{aligned} \alpha &= \frac{1}{2}\left(b + c + \sqrt{(b-c)^2 + 4a^2}\right), \\ \beta &= \frac{1}{2}\left(b + c - \sqrt{(b-c)^2 + 4a^2}\right), \end{aligned} \tag{12}$$

Introducing $\cos\theta \equiv \langle\mathbb{T}|\alpha\rangle/\delta\bar{T}$, Eq. (11) takes form

$$P_{max} = \frac{1}{4}(\alpha\cos^2\theta + \beta\sin^2\theta)\delta\bar{T}^2 \tag{13}$$

with $\delta\bar{T} = \sqrt{(\delta T_1)^2 + (\delta T_2)^2}$ and the expression in the brackets defines three-terminal power factor PF

$$\mathrm{PF} = (\alpha\cos^2\theta + \beta\sin^2\theta) \tag{14}$$

where θ is explicitly expressed as $\cos\theta = (\delta T_1\alpha_1 + \delta T_2\alpha_2)/(\delta\bar{T}\sqrt{\langle\alpha|\alpha\rangle})$ with $|\alpha\rangle = [-a/(c-\alpha), 1]$.

Independent contribution of tunneling processes to conductance

There are four competing tunneling processes that contribute to conductance G_{ij}, indicated by SP, QP, DAR, and CAR and introduced in the main article. Therefore, for reference, here we present the contributions to electrical conductance due to individual tunneling processes for varied system parameters.

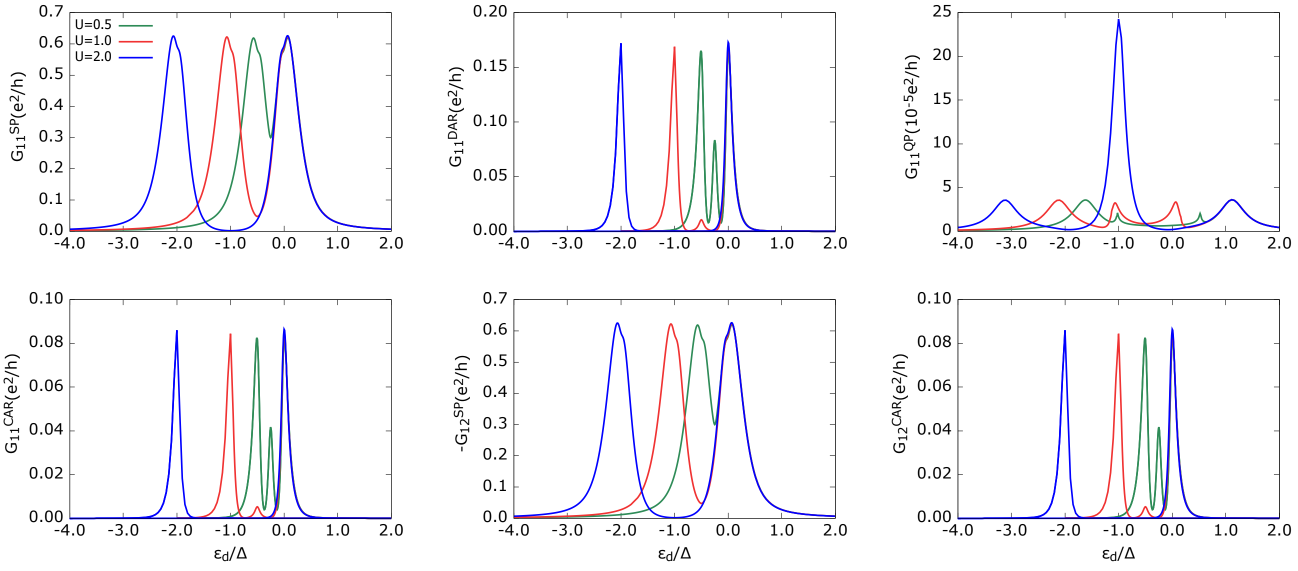

Figure S1. Intra-dot Coulomb correlation factor (U) dependence of four contributions to the electrical conductance. Assumed parameters: p = 0.5, R = 1, A = 1, Γ = 0.1Δ, k_bT = 0.1Δ, corresponds to those in Fig.2 in the main text.

As seen in Fig. S1, the outermost resonance peak intensities, for $G^{\mathrm{X}}_{ij}\, i,j = 1,2$ and X= SP, DAR, CAR, QP,s remain unchanged with varying U and manifestly $G^{\mathrm{X}}_{1i} = G^{\mathrm{X}}_{2i}$. For CAR and DAR conductance, the resonance at the particle hole symmetry point ($\varepsilon_d = -U/2$) decreases with increasing U. This resonance is due to Andreev processes occurring through the $-U/2$ and $U/2$ levels (corresponding to the density of states maxima). When U increases, these levels move away from the Fermi level of leads, resulting in the reduction of both DAR and CAR Andreev tunneling processes.

Notice also that under this parametric condition, $G^{\mathrm{DAR}}_{ii} = 2G^{\mathrm{CAR}}_{ii}$. In general, quasiparticle contribution to the electrical conductance reveals four peaks. These maxima correspond to the resonances when ε_d or $\varepsilon_d + U$ crosses (are slightly above/below) the SC gap edges at $\pm\Delta$, i. e. when $\varepsilon_d = \pm\Delta$ and $\varepsilon_d = \pm\Delta - U$. The three-peak structure for $U = 2\Delta$ origins from the imposition of resonances at $\varepsilon_d = -\Delta$ and $\varepsilon_d = \Delta - U$.

The change in coupling of FM_2 lead, by varying parameter A, brings different qualitative changes in the conductance components compared to U variation. In Fig. S2, the observation of $G^{\mathrm{SP,CAR}}_{ij} = G^{\mathrm{SP,CAR}}_{ji}$, $i = 1,2$ ensures preservation of the time-reversal symmetry of nonlocal conductance. G^{CAR}_{ij} increases as A is varied from 0.01 to ≈ 1 until saturation and decreases for $A > 1$. The coupling variation of FM_2 differently influences $G^{\mathrm{DAR,QP}}_{11}$ and $G^{\mathrm{DAR,QP}}_{22}$.

The amplitude of the $G^{\mathrm{DAR,QP}}_{11}$ peaks decreases with A, while those of $G^{\mathrm{DAR,QP}}_{22}$ reveal opposite behavior. The latter dependence is rather clear as $G^{\mathrm{DAR,QP}}_{22}$ directly depends on Γ_2, e. g. recall that $G^{\mathrm{DAR}}_{22\sigma} = \Gamma^{\sigma}_2\Gamma^{\bar{\sigma}}_2|G^r_{12}|^2$. Thus, for small A, not only DAR and QP but also CAR and SP processes are inhibited. In turn, the former situation is more complex as e. g. $G^{\mathrm{DAR}}_{11\sigma} = \Gamma^{\sigma}_1\Gamma^{\bar{\sigma}}_1|G^r_{12}|^2$ does not depend directly on Γ_2. As mentioned in the main article, here, an increase of DAR tunneling processes occurs in favor of SP events as A becomes gradually smaller. This phenomenon can be supported by the following equations obtained for maxima of G^{DAR}_{ii} for $T = 0$, $\varepsilon_d = 0$, and $R = 1$,

$$G^{\mathrm{DAR}}_{11} = \frac{4e^2}{h}\frac{4(1-p^2)}{[(1-p^2)(1+A)^2+1]^2}, \tag{15a}$$

$$G^{\mathrm{DAR}}_{22} = A^2 G^{\mathrm{DAR}}_{11} \tag{15b}$$

which clearly shows that G_{11}^{DAR} monotonically decreases with increasing A. In turn, for $G_{22}^{\mathrm{DAR,QP}}$, the resonance amplitude is greater for larger A. Further, an interesting feature observed is that for a given finite A, the full width at half maximum (FWHM) of $G_{11}^{\mathrm{DAR,QP}}$'s peaks is equal to that of $G_{22}^{\mathrm{DAR,QP}}$.

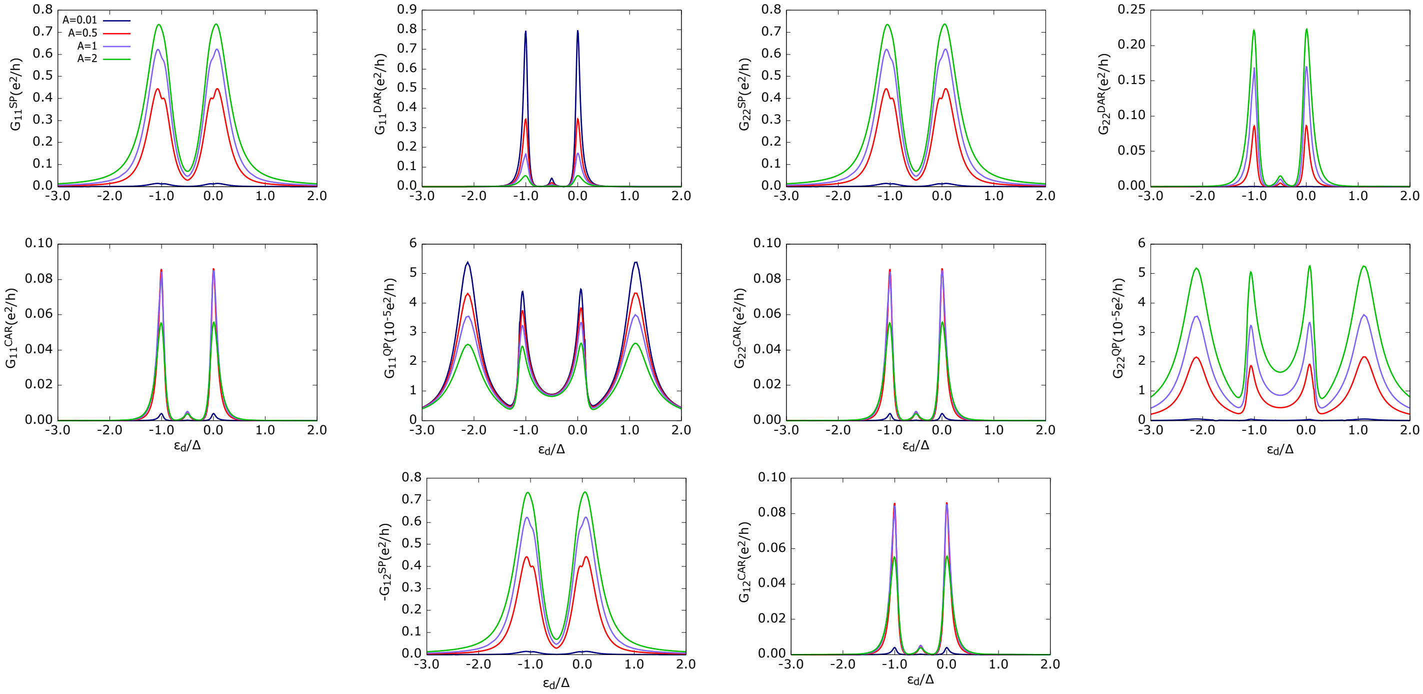

Figure S2. FM_2 coupling (Γ_2) dependence of four contributions to the electrical conductance. Parameters: U=Δ, Other parameters are same as Fig. S1, corresponds to Fig.3 in the main text.

Analyzing the influence of spin polarization of FM leads, the SP conductance for parallel configuration in Fig. S3 remains unchanged by the variation of p. Since here, the density of states for individual spin in two FM leads overlap perfectly (thus $G_{ij\uparrow(\downarrow)}^{\mathrm{SP}}$ varies but G_{ii}^{SP} is constant.) The decreasing of G_{ii}^{QP} with increasing p can be understood on similar ground. The overlap of density of state (DOS) for majority (spin-up) carriers in FM electrode with DOS for spin-up electrons in SC lead though remains unchanged with increasing p, the relevant flux of minority carriers (spin-down electrons) decreases resulting in decreasing electrical conductance. In turn, SP and QP tunneling processes is reduced in AP alignment with increasing p. In both magnetic configurations, DAR processes are suppressed with increasing p, whereas CAR processes are attenuated (enhanced) in P (AP) alignment. For an explanation, we refer to the main text.

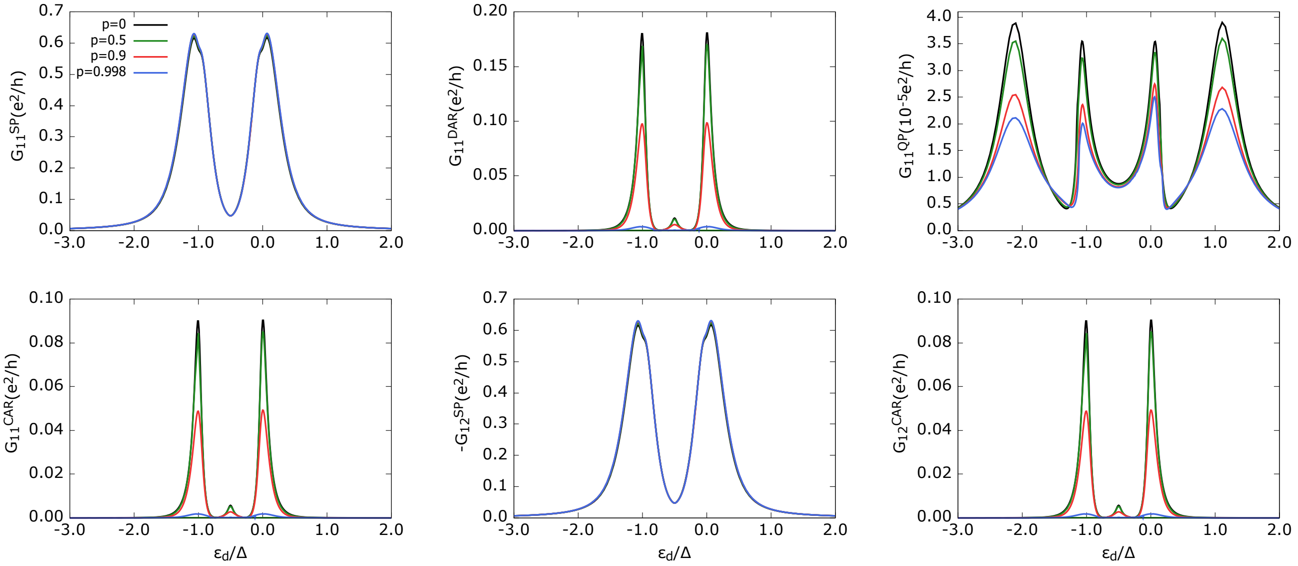

Figure S3. Spin polarization p dependence of four contributions to the electrical conductance for parallel magnetic configuration. Other parameters same as in Fig. S1, corresponds to those in Fig.5 in the main text.

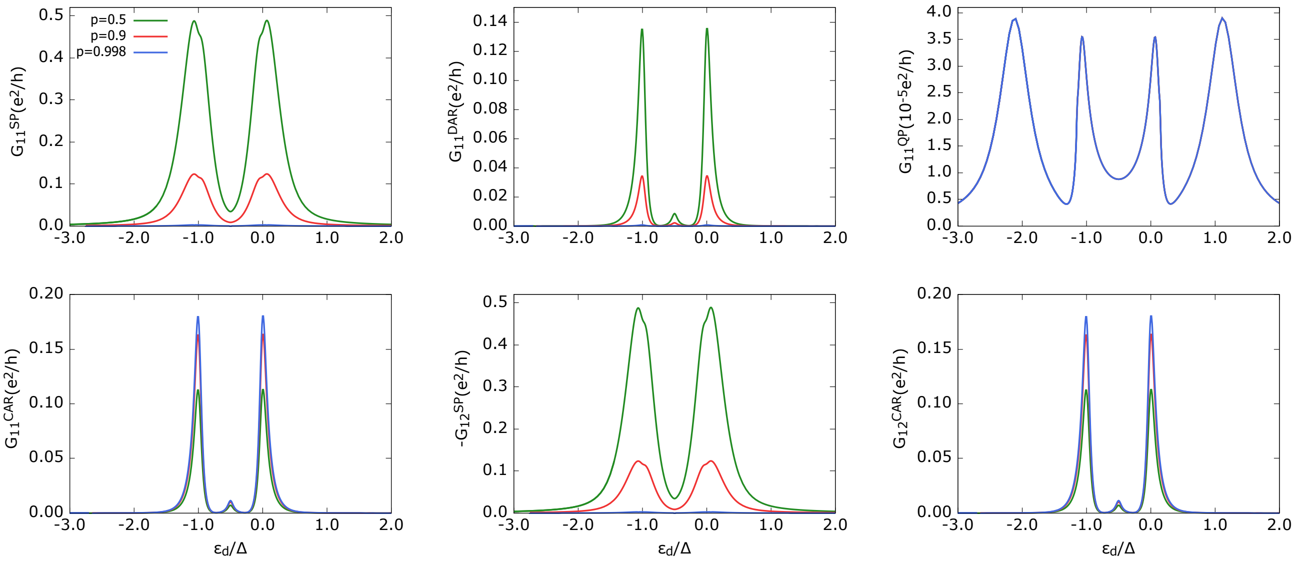

Figure S4. Spin polarization p dependence of four contributions to the electrical conductance for antiparallel magnetic configuration. Other parameters same as in Fig. S1, corresponds to those in Fig.5 in the main text.

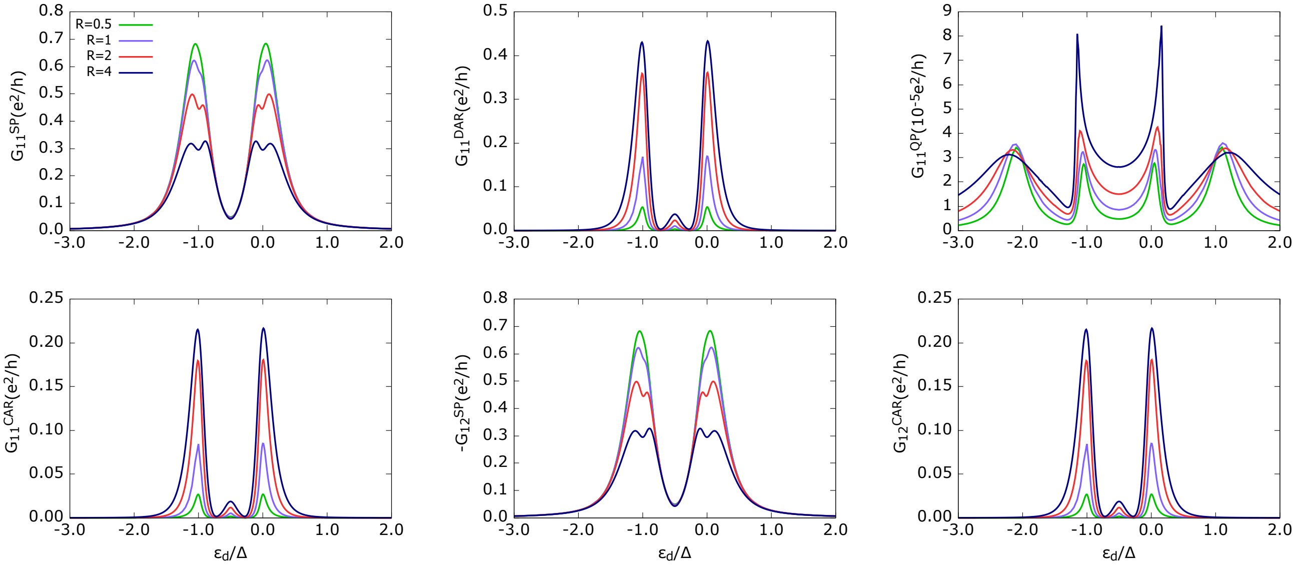

Figure S5. Superconductor coupling (Γ_3) dependence of four contributions to the electrical conductance. Other parameters same as in Fig. S1, corresponds to those in Fig.4 in the main text.

The decreasing (increasing) trend of SP (CAR, DAR) conductance with SC coupling is clearly understood from Fig. S5. The behavior of DAR and CAR conductance's amplitude can be, to some extent, explained by considering simplified formulae obtained in a similar way as Eq. (15), but for $A = 1$,

$$G_{ii}^{\mathrm{DAR}} = \frac{4e^2}{h}\frac{4R^2(1-p^2)}{[4(1-p^2)+R^2]^2}, \tag{16a}$$

$$G_{ij}^{\mathrm{CAR}} = \frac{1}{2}G_{ii}^{\mathrm{DAR}}. \tag{16b}$$

Note also that width of the maxima of G_{ij}^{X} become larger with increasing R, except inner peaks of the G_{ij}^{QP}. In particular, the widths of the Andreev related peaks strongly depend on the coupling to the SC electrode, which is opposite to the behavior found in the large SC gap ($\Delta \to \infty$)[3–5]. This becomes evident when comparing SC self-energy, given by Eq. (8) in the main text, with its large gap limiting case which acquires the form,

$$\mathbf{\Sigma}^r_{3\Delta\to\infty} = \frac{\Gamma_3}{2}\begin{pmatrix} 0 & 1 & 0 & 0 \\ 1 & 0 & 0 & 0 \\ 0 & 0 & 0 & -1 \\ 0 & 0 & -1 & 0 \end{pmatrix}. \tag{17}$$

Self-energy (17) is real, and thus, only affects dot's level position without influencing its width.

The G_{ii}^{QP} trend is intriguing. While the peaks outside of the gap become blunt and wide, the subgap peaks in G_{ii}^{QP} exhibit an increasing tendency with a larger R. This broadening of outer peaks with R results from the quantum dot's level broadening in relation to the superconductor's accessible density of states. The subgap behavior, however, reinforces the finding about Fig. S1, which states that these peaks exist because the resonance condition for a sharp value of ε_d is satisfied.

Effect of temperature–supplementary figure

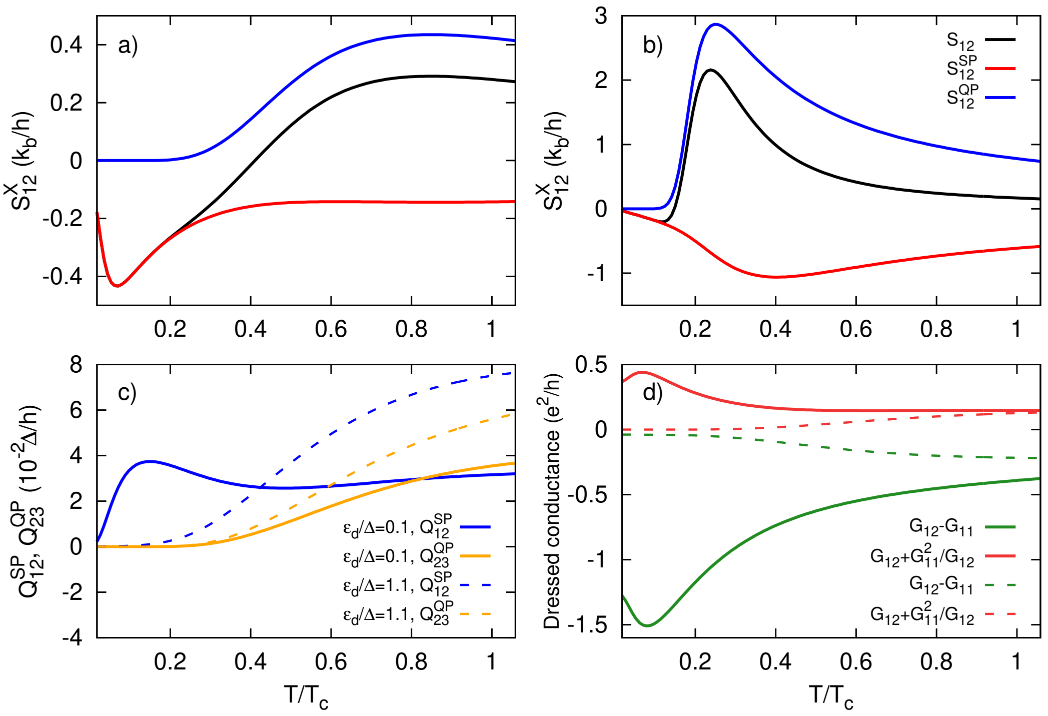

Figure S6. Temperature dependence of Seebeck coefficient S_{12} and its components: S_{12}^{SP} and S_{12}^{QP} calculated for a) $\varepsilon_d/\Delta = 0.1$, b) $\varepsilon_d/\Delta = 1.1$, the corresponding c) heat transport coefficients Q_{12}^{SP} and Q_{23}^{QP}, d) dressed conductance. Solid (dotted) lines in c) and d) correspond to $\varepsilon_d/\Delta = 0.1$ ($\varepsilon_d/\Delta = 1.1$). Temperature is measured in the units of SC critical temperature T_c.

Heat conductance in spin thermoelectricity–supplementary figure

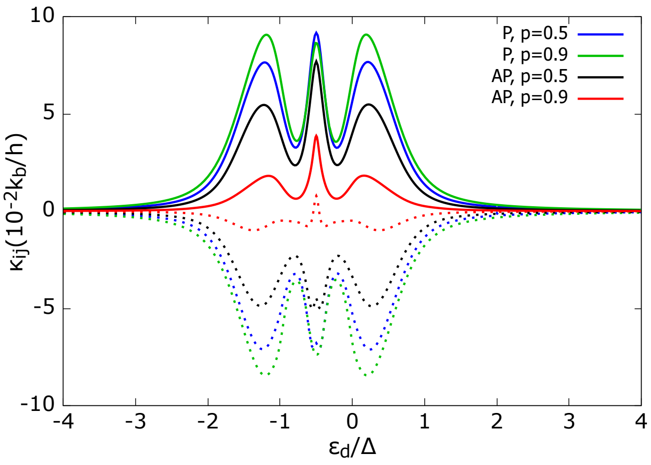

Figure S7. Heat conductance κ calculated for parallel (P) and antiparallel (AP) magnetic configuration of FM leads, and for indicated values of spin-polarization factor p. Solid and dotted lines indicate κ_{11} and κ_{12} respectively. Other parameters same as corresponding to Fig.7 in the main text.

References

1. Trocha, P. & Barnaś, J. Spin-polarized Andreev transport influenced by Coulomb repulsion through a two-quantum-dot system. *Phys. Rev. B* **89**, 245418, 10.1103/PhysRevB.89.245418 (2014).
2. Verma, S. & Singh, A. Non-equilibrium thermoelectric transport across normal metal–quantum dot–superconductor hybrid system within the Coulomb blockade regime. *J. Physics: Condens. Matter* **34**, 155601, 10.1088/1361-648X/ac4ced (2022).
3. Weymann, I. & Trocha, P. Superconducting proximity effect and zero-bias anomaly in transport through quantum dots weakly attached to ferromagnetic leads. *Phys. Rev. B* **89**, 115305, 10.1103/PhysRevB.89.115305 (2014).
4. Trocha, P. & Weymann, I. Spin-resolved Andreev transport through double-quantum-dot Cooper pair splitters. *Phys. Rev. B* **91**, 235424, 10.1103/PhysRevB.91.235424 (2015).
5. González I., A., Pacheco, M., Calle, A. M., Siqueira, E. C. & Orellana, P. A. Dicke and Fano-Andreev reflections in a triple quantum-dot system. *Sci. Reports* **11**, 3941, 10.1038/s41598-021-83407-6 (2021).
